# USAG-1 aggravates renal ischemia‒reperfusion injury via promoting GPX4 degradation-induced ferroptosis

**DOI:** 10.1038/s41419-026-08904-w

**Published:** 2026-05-23

**Authors:** Xiaohu Li, Huimeng Wang, Hongxuan Ma, Jiajia Sun, Yongsheng Luo, Minghui Qin, Hao Zhang, Jinfeng Li

**Affiliations:** Department of Kidney Transplantation, The First Affiliated Hospital of Zhengzhou University, Zhengzhou, China

**Keywords:** Mechanisms of disease, Acute kidney injury

## Abstract

Renal ischemia‒reperfusion injury (IRI) remains an inevitable complication in kidney transplantation and a leading cause of delayed graft function (DGF). Ferroptosis, a form of iron-dependent lipid peroxidation-driven cell death, has emerged as an important mechanism contributing to renal IRI. Although uterine sensitization-associated gene-1 (USAG-1) has been implicated in both acute and chronic kidney injury, its involvement in IRI-associated ferroptosis has not been elucidated. In this study, using murine renal ischemia–reperfusion models and human transplant kidney biopsies (from donation after circulatory death donors), we demonstrate that USAG-1 is significantly upregulated under ischemic stress. Importantly, higher USAG-1 expression in donor kidneys was associated with worse allograft function post-transplantation, suggesting that USAG-1 may serve as a promising biomarker for transplant injury and outcomes. In addition, genetic ablation of USAG-1 markedly attenuated both IRI- and folic acid-induced ferroptosis, accompanied by reduced acute kidney injury severity. Mechanistically, glutathione peroxidase 4 (GPX4) was a critical downstream effector of USAG-1 in mediating ferroptotic processes. HSP family A member 5 (HSPA5), a canonical molecular chaperone, stabilized GPX4 via direct interactions. Our findings revealed that overexpressed USAG-1 competitively binds to HSPA5, thereby disrupting the HSPA5–GPX4 interaction. This interference was abrogated by truncation mutants of USAG-1 that failed to interact with HSPA5. Notably, functional validation of the USAG-1/HSPA5/GPX4 axis confirmed that preserving HSPA5–GPX4 binding mitigated ferroptosis and alleviated renal injury. Overall, our study reveals a previously unrecognized mechanism by which USAG-1 promotes ferroptosis in renal IRI and highlights the therapeutic potential of targeting the USAG-1/HSPA5/GPX4 axis to improve graft outcomes post-transplantation.

## Introduction

Kidney transplantation has become an important therapeutic option for patients with end-stage renal disease [1]. However, ischemia‒reperfusion injury (IRI) during donor maintenance, procurement, and transplantation often leads to delayed graft function (DGF), which adversely affects the long-term survival of the allograft [2, 3]. With the growing shortage of donor organs, the use of expanded criteria donors and donation after circulatory death (DCDs) has increased in clinical practice [4, 5]. Nevertheless, these types of donor kidneys are prone to more severe IRI, which leads to a significantly higher incidence of DGF and an increased risk of graft failure [4, 6, 7]. Unfortunately, there are currently no specific molecular interventions targeting IRI [8], highlighting the urgent need to develop novel therapeutic strategies to reduce the incidence of DGF following high-risk kidney transplantation and to improve long-term allograft outcomes.

The mechanisms underlying parenchymal cell injury caused by IRI are highly complex and involve necrosis, apoptosis, and other forms of programmed cell death mediated by oxidative stress [9–11]. Recent studies have identified ferroptosis, a form of iron-dependent regulated cell death, as a key contributor to renal IRI [12]. Ferroptosis is characterized by the iron- and reactive oxygen species (ROS)-mediated accumulation of lipid peroxides and is morphologically and biochemically distinct from apoptosis and necrosis [13–15]. Growing evidence suggests that ferroptosis serves as a major driver of early injury during IRI, where the initial wave of cell death triggers an amplification of inflammatory damage [12, 16, 17]. In renal IRI models, ferroptosis-associated mitochondrial alterations—such as increased membrane density and a reduction in or loss of cristae—along with endoplasmic reticulum swelling have been observed. Importantly, treatment with ferroptosis inhibitors (e.g., liproxstatin-1) significantly reverses this ultrastructural damage and protects renal tubular cells [18]. Therefore, ferroptosis is increasingly recognized as a promising therapeutic target in the fields of acute kidney injury (AKI) and IRI.

Uterine sensitization-associated gene-1 (USAG-1), encoded by the *SOSTDC1* gene, is a secreted glycoprotein containing a coiled-coil domain and belongs to the bone morphogenetic protein (BMP) antagonist family [19]. Initially identified in the endometria of sensitized rats, USAG-1 was later found to be highly expressed in the kidney, where it plays a critical role in renal development and disease by modulating both the BMP and Wnt signaling pathways [20]. USAG-1 directly binds to BMPs—particularly BMP7—and inhibits their interaction with receptors, thereby suppressing BMP-mediated signaling [21, 22]. In models of kidney disease, USAG-1 has been shown to influence the progression of both acute and chronic renal injury and the recovery of graft function after kidney transplantation [23, 24]. In addition, USAG-1 is involved in immune regulation by suppressing B cell responses within lymph node germinal centers and reducing humoral immune activity, suggesting potential therapeutic implications for autoimmune kidney diseases and antibody-mediated rejection following kidney transplantation [21, 25]. Despite the growing interest in the role of USAG-1 in BMP signaling and immune modulation, its specific function in the pathogenesis of renal IRI and the underlying molecular mechanisms remain largely unclear.

In this study, through a murine renal IRI model and clinical donor kidney samples from DGF cases, we found that USAG-1 expression is significantly upregulated under ischemic conditions and positively correlated with the severity of ischemia. These results suggest that USAG-1 may serve as a key regulatory factor in the pathogenesis of IRI. To further explore this, we employed a combination of transcriptomic, proteomic, and transgenic mouse approaches and, for the first time, demonstrated that USAG-1 exerted a detrimental effect during renal IRI. Specifically, we revealed a novel molecular mechanism whereby USAG-1 competitively bound to the heat shock protein HSPA5, thereby disrupting the HSPA5-mediated stabilization of GPX4, promoting ferroptosis in renal tubular epithelial cells, and exacerbating IRI. These findings provide new insights and potential therapeutic targets for the prevention and treatment of renal transplantation-related IRI.

## Results

### Renal ischemia upregulates the expression of USAG-1 in renal tubular epithelial cells, an effect correlated with poor graft function and DGF in kidney transplantation

**Fig. 1:**
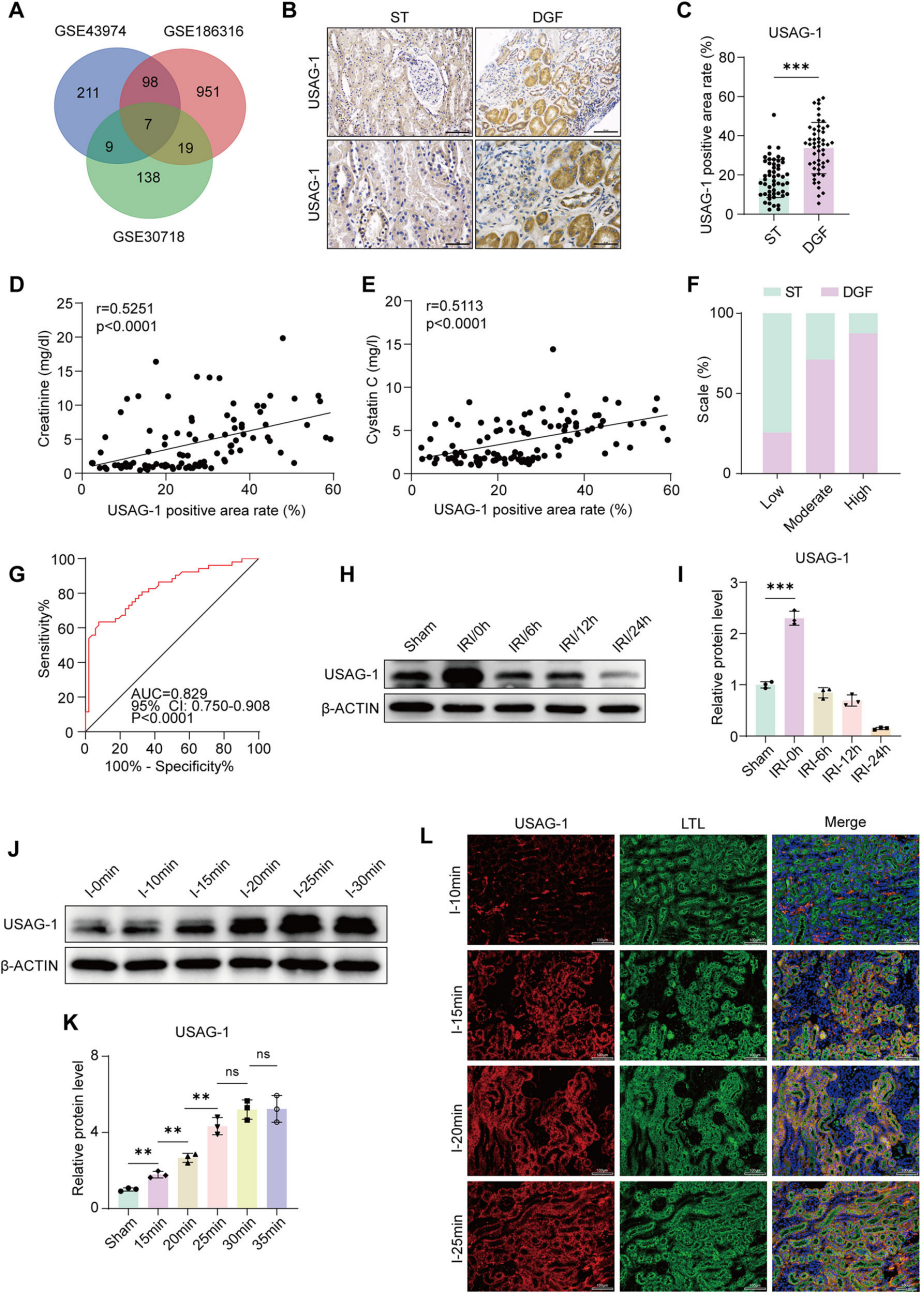
Upregulation of USAG-1 expression in tubular epithelial cells following renal ischemia predicts poor allograft function and DGF. **A** Venn diagram of differentially expressed genes identified from the GEO datasets GSE43974, GSE30718, and GSE186316. **B**, **C** Representative immunohistochemical staining of USAG-1 in time-zero kidney biopsy specimens from DCD donor kidneys in the stable function (ST, *n* = 52) and delayed graft function (DGF, *n* = 52) groups, with quantification based on the USAG-1-positive area(scale bars: 100 μm and 50 μm). **D**, **E** Spearman correlation analyses between the USAG-1-positive area in donor kidney biopsy specimens and recipient Scr and cystatin C levels at postoperative week 1. **F** DGF incidence stratified by USAG-1 expression levels in donor kidneys. **G** ROC curve analysis of the ability of donor USAG-1 expression to predict DGF. **H**, **I** Western blot analysis and quantification of USAG-1 protein expression in whole-kidney lysates from mice subjected to renal IRI with different reperfusion time points after ischemia. **J**, **K** Western blot analysis and quantification of USAG-1 protein expression in whole-kidney lysates from mice subjected to different durations of ischemia with 0 h reperfusion. **L** Representative immunofluorescence (IF) staining of USAG-1 (red), LTL (green), and DAPI (blue) in mouse kidneys after different durations of ischemia (scale bar: 100 μm). ns: *p* > 0.05; *p < 0.05; **p < 0.01; ***p < 0.001.

To identify genes associated with AKI, we analyzed differentially expressed genes from the GSE43974, GSE30718, and GSE186316 datasets in the GEO database. A Venn diagram revealed seven common differentially expressed genes (Fig. 1A), and the complete list of these candidates is provided in Supplemental Table S1. Among them, the kidney-specific expression pattern of USAG-1 particularly attracted our attention. While USAG-1 has been linked to kidney injury and repair, its role in IRI remains unclear. IRI is an inevitable complication of kidney transplantation, particularly severe in DCD kidneys due to prolonged warm ischemia. This exacerbates oxidative stress, inflammation, and microvascular injury, significantly increasing DGF incidence and impairing long-term graft survival. To clarify the clinical relevance of USAG-1 in transplantation-related IRI, we analyzed time-zero kidney biopsies from DCD donors with DGF or stable function (ST). Our analysis demonstrated significantly higher USAG-1 expression in kidneys from the DGF group compared to the ST group (Fig. 1B, C). Further analysis of posttransplant clinical data revealed that donor kidneys with high USAG-1 expression were associated with poorer renal function and higher DGF incidence post-transplantation (Fig. 1D‒F). Comparison of donor and recipient baseline characteristics between groups, along with univariate and multivariate logistic regression analyses, confirmed that high donor renal USAG-1 expression was an independent risk factor for DGF (Supplemental Tables S2‒S4). Receiver operating characteristic (ROC) curve analysis also revealed that USAG-1 expression in donor kidneys effectively predicted DGF occurrence (Fig. 1G). Collectively, these findings suggest that USAG-1 expression during ischemia in kidney grafts may serve as an indicator of donor kidney quality and is potentially predictive of graft recovery outcomes post-transplantation.

Next, we explored the role of USAG-1 using a mouse model of renal IRI. We found that USAG-1 expression was markedly elevated during the ischemic phase and gradually declined with increasing reperfusion time (Fig. 1H, I). To further examine its association with ischemic severity, we subjected mice to varying durations of ischemia and collected kidney tissues immediately upon restoration of normal organ color. Western blot analysis and immunofluorescence staining revealed that USAG-1 expression increased progressively with prolonged ischemia time (Fig. 1J, K). Notably, co-localization with Lotus tetragonolobus lectin (LTL), a marker of renal tubular epithelium, revealed that the upregulation of USAG-1 predominantly occurred in tubular epithelial cells, indicating that these cells are the primary site of USAG-1 accumulation during ischemic injury (Fig. 1L). To further define the tubular segment in which USAG-1 was upregulated after ischemic injury, we performed additional co-immunostaining with markers of different nephron segments, including NKCC2, AQP2, and Calbindin-D28K. Compared with the prominent overlap observed with LTL, USAG-1 showed much less co-localization with these markers, suggesting that the increased USAG-1 signal after ischemia is most consistent with proximal tubular localization (Supplementary Fig. S1A).

Together, these findings highlight USAG-1 as a potential early marker of ischemic injury in renal tubules and a candidate target for assessing ischemic burden in kidney grafts.

### USAG-1 deficiency alleviates IRI-induced AKI

**Fig. 2:**
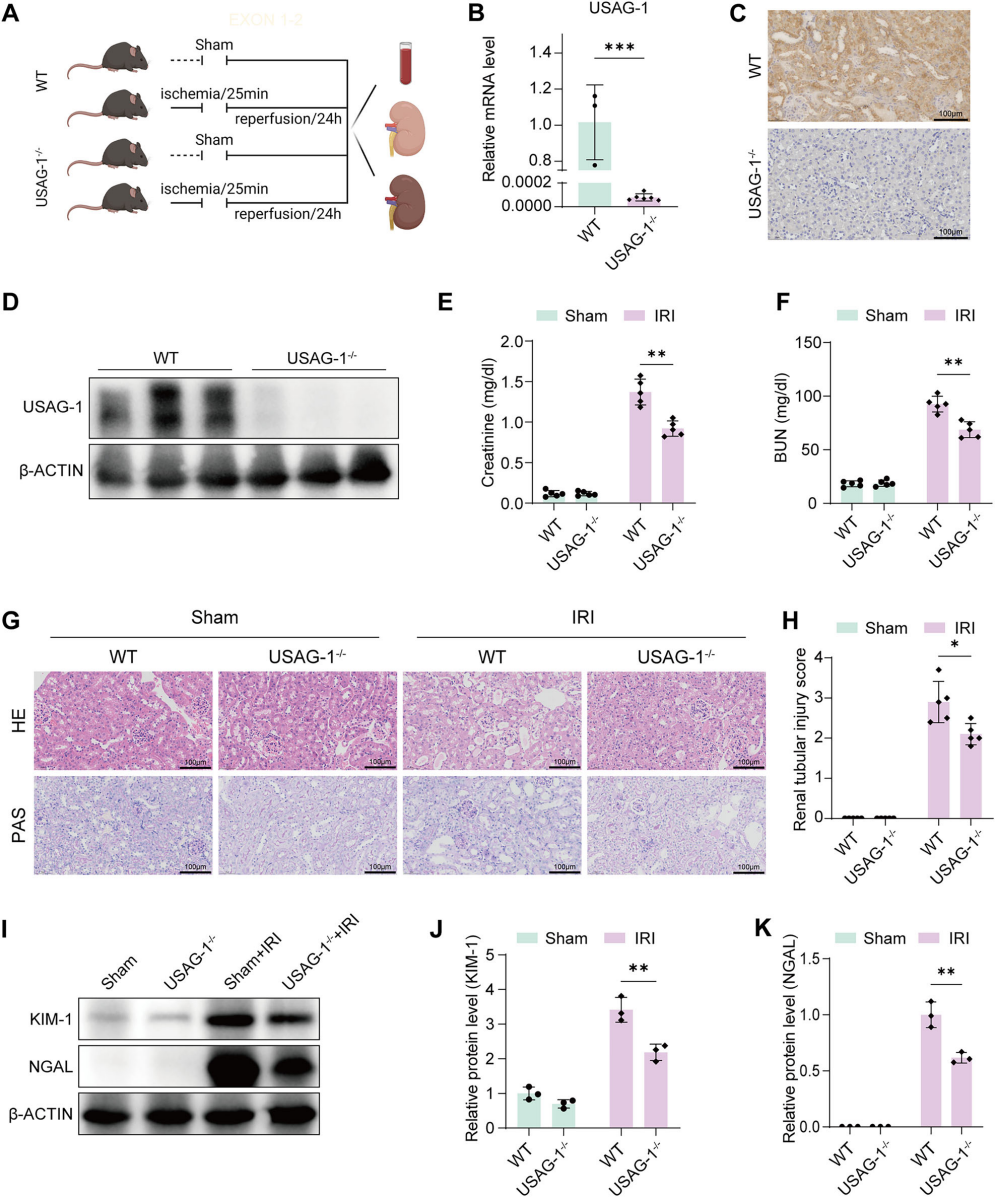
USAG-1 deficiency attenuates renal injury following ischemia–reperfusion injury (IRI). **A** Schematic diagram of the experimental groups. **B**–**D** Validation of renal USAG-1 deletion in WT and USAG-1-KO mice by RT-qPCR and Western blotting using whole-kidney tissues/lysates, and by immunohistochemistry on kidney sections. **E**, **F** Scr and BUN levels in WT and USAG-1^−/−^ mice subjected to sham or IRI treatment. **G**, **H** Representative H&E and PAS staining of kidney sections and tubular injury scores (scale bar, 50 μm; *n* = 5). **I**–**K** Western blot analysis and quantification of KIM-1 and NGAL expression in whole-kidney lysates (*n* = 3). ns: *p* > 0.05; *p < 0.05; **p < 0.01; ***p < 0.001.

Previous studies have reported that USAG-1 plays a negative regulatory role in several models of drug-induced AKI [21]. Our data further revealed that USAG-1 expression is significantly altered in the context of IRI; however, its precise role in IRI remains unclear. To further explore the function of USAG-1 under pathological conditions, USAG-1 global knockout (USAG-1^−/−^) mice were subjected to renal IRI (Fig. 2A). RT-qPCR, Western blotting, and immunohistochemistry of whole-kidney tissues confirmed efficient renal deletion of USAG-1 in USAG-1^−/−^ mice (Fig. 2B‒D). We then subjected both wild-type (WT) and USAG-1^−/−^ mice to renal IRI or sham surgery. No significant differences were observed between the sham-WT and sham-USAG-1^−/−^ groups in serum creatinine (Scr), blood urea nitrogen (BUN) (Fig. 2E, F), or histological scores (Fig. 2G, H), indicating that USAG-1 deletion does not affect baseline renal function. In contrast, following IRI, USAG-1^−/−^ mice exhibited significantly improved renal function (lower Scr and BUN levels; Fig. 2E, F) and reduced histopathological injury, as demonstrated by hematoxylin–eosin (HE) and periodic acid-Schiff (PAS) staining (Fig. 2G, H). Moreover, the expression of renal injury markers KIM-1 and NGAL was markedly decreased in USAG-1^−/−^ mice, indicating attenuation of IRI-induced kidney damage (Fig. 2I–K).

To further elucidate the role of USAG-1 in IRI-induced AKI, we generated mice with renal tubular epithelial cell-specific overexpression of USAG-1 via adenoviral injection, followed by induction of IRI (Supplementary Fig. S2A). Successful overexpression was confirmed by Western blot analysis and GFP fluorescence imaging (Supplementary Fig. S2B, C). Compared with control mice, IRI-ad-USAG-1 mice exhibited worse renal function, as evidenced by increased blood Scr and BUN levels (Supplementary Fig. S2D, E), along with more severe histological damage observed via HE and PAS staining (Supplementary Fig. S2F, G). Consistent with these findings, the expression of KIM-1 and NGAL was markedly increased in the kidneys of USAG-1–overexpressing mice (Supplementary Fig. S2H–J), further supporting the aggravating effect of USAG-1 on IRI-induced renal injury.

Collectively, these results indicate that USAG-1 acts as a negative regulator in the context of IRI: its genetic deletion confers protection against renal injury, whereas its overexpression exacerbates IRI-induced kidney damage.

### USAG-1 modulates ferroptosis in HK-2 cells under hypoxia-reoxygenation stress

**Fig. 3:**
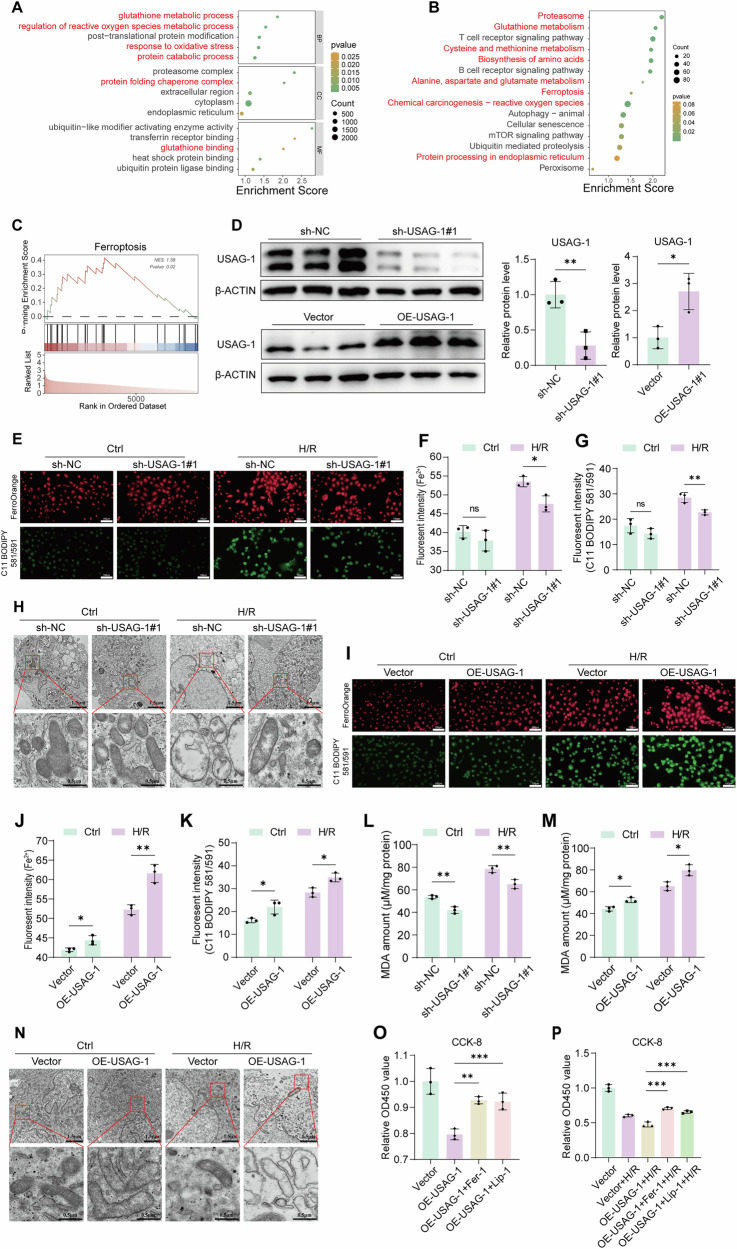
USAG-1 knockdown alleviates and USAG-1 overexpression exacerbates H/R-induced ferroptosis in HK-2 cells. **A**, **B** GO enrichment analysis and KEGG pathway enrichment analysis of differentially expressed proteins from proteomic profiling. **C** GSEA of the proteomic dataset showing enrichment of ferroptosis-related pathways. **D** Western blot validation of stable USAG-1 knockdown and transient USAG-1 overexpression efficiency in HK-2 cells. **E**–**G** After stable USAG-1 knockdown with sh-USAG-1#1, intracellular Fe²⁺ and lipid ROS levels were measured in HK-2 cells under Control or H/R conditions. Representative fluorescence images of intracellular Fe²⁺ detected by FerroOrange and lipid ROS detected by C11 BODIPY 581/591 are shown in (**E**), with quantification in (**F**) and (**G**), respectively. (scale bar: 100 μm; *n* = 3). **H** Representative TEM images showing mitochondrial ultrastructural changes in sh-NC- and sh-USAG-1#1-transduced HK-2 cells under control or H/R conditions. **I**–**K** After transient USAG-1 overexpression, intracellular Fe²⁺ and lipid ROS levels were measured in HK-2 cells under Control or H/R conditions. Representative fluorescence images of intracellular Fe²⁺ detected by FerroOrange and lipid ROS detected by C11 BODIPY 581/591 are shown in (**I**), with quantification in (**J**) and (**K**), respectively. **L** Measurement of MDA levels in sh-NC- and sh-USAG-1#1-transduced HK-2 cells under Control or H/R conditions. **M** Measurement of MDA levels in vector- and OE-USAG-1-transfected HK-2 cells under Control or H/R conditions. **N** Representative TEM images showing mitochondrial ultrastructural changes in vector- and OE-USAG-1-transfected HK-2 cells under control or H/R conditions. **O**, **P** HK-2 cells were cultured in vitro, transfected with a USAG-1 overexpression plasmid, treated with ferroptosis inhibitors, and subjected to H/R; cell viability was assessed by CCK-8 assays. ns : *p* > 0.05; *p < 0.05; **p < 0.01; ***p < 0.001.

To elucidate the molecular mechanism of USAG-1 in renal IRI, we overexpressed USAG-1 in human renal proximal tubular epithelial (HK-2) cells and conducted multi-omics profiling. Principal component analysis (PCA) clearly distinguished USAG-1–overexpressing cells from controls (Supplemental Fig. S3A, F), with volcano plots and heatmaps revealing distinct transcriptional (Supplemental Fig. S3B, C) and proteomic signatures (Supplemental Fig. S3G, H). Functional enrichment analysis of the transcriptomic dataset is shown in Supplemental Fig. S3D, E, whereas the enrichment analyses shown in Fig. 3A, B were derived from the proteomic dataset. Notably, both datasets converged on pathways related to glutathione metabolism, cysteine utilization, and ferroptosis. Critically, gene set enrichment analysis (GSEA) confirmed the pronounced dysregulation of ferroptosis-related pathways (Fig. 3C), identifying USAG-1 as a key regulator of iron-dependent cell death during IRI.

Ferroptosis is a newly recognized form of regulated cell death driven by iron-dependent lipid peroxidation [26]. Accumulating evidence has established ferroptosis as a major mechanism of early cell death in renal IRI, contributing substantially to tissue damage [27]. To investigate the potential link between USAG-1 and ferroptosis, we employed an in vitro hypoxia-reoxygenation (H/R) model in HK-2 cells, in which stable USAG-1 knockdown was achieved by lentiviral shRNA transduction and USAG-1 overexpression was achieved by transient plasmid transfection; the efficiency of these modifications was confirmed by Western blotting (Fig. 3D). Upon H/R induction, functional assays showed that USAG-1 knockdown significantly reduced ferroptosis markers, including ferrous iron (Fe²⁺) accumulation (Fig. 3E, F), lipid ROS production (Fig. 3E, G), and malondialdehyde (MDA) levels (Fig. 3L), indicating that USAG-1 depletion mitigates ferroptotic injury under H/R stress. Transmission electron microscopy further revealed that H/R-induced ferroptosis-associated mitochondrial ultrastructural alterations, including mitochondrial shrinkage, increased membrane density, and reduced or lost cristae, were markedly alleviated by USAG-1 knockdown (Fig. 3H). To further exclude potential off-target effects, we performed parallel validation using a second independent shRNA targeting USAG-1 (sh-USAG-1#2). Consistent with the results obtained using the original knockdown construct, sh-USAG-1#2 effectively reduced USAG-1 protein expression and similarly attenuated H/R-induced increases in MDA, intracellular Fe²⁺, and lipid ROS levels in HK-2 cells (Supplementary Fig. S4A–F). TEM analysis further supported these findings by showing that sh-USAG-1#2 alleviated the H/R-induced mitochondrial ultrastructural abnormalities characteristic of ferroptosis (Supplementary Fig. S4G). Conversely, USAG-1 overexpression in HK-2 cells (Fig. 3D) led to a marked increase in Fe²⁺ accumulation, lipid ROS levels (Fig. 3I–K), and MDA content (Fig. 3M), thereby aggravating H/R-induced ferroptosis. Consistently, TEM analysis showed that USAG-1 overexpression further exacerbated H/R-induced mitochondrial ultrastructural damage (Fig. 3N). Importantly, treatment with ferroptosis inhibitors ferrostatin-1 (Fer-1) and liproxstatin-1 (Lip-1) effectively rescued cell viability in USAG-1–overexpressing cells (Fig. 3O, P). Collectively, these findings demonstrate that USAG-1 promotes ferroptosis in renal tubular epithelial cells and may contribute to IRI-associated tubular injury through the modulation of iron-dependent oxidative stress pathways.

### USAG-1 regulates ferroptosis of renal tubular epithelial cells in both IRI and folic acid-induced AKI

**Fig. 4:**
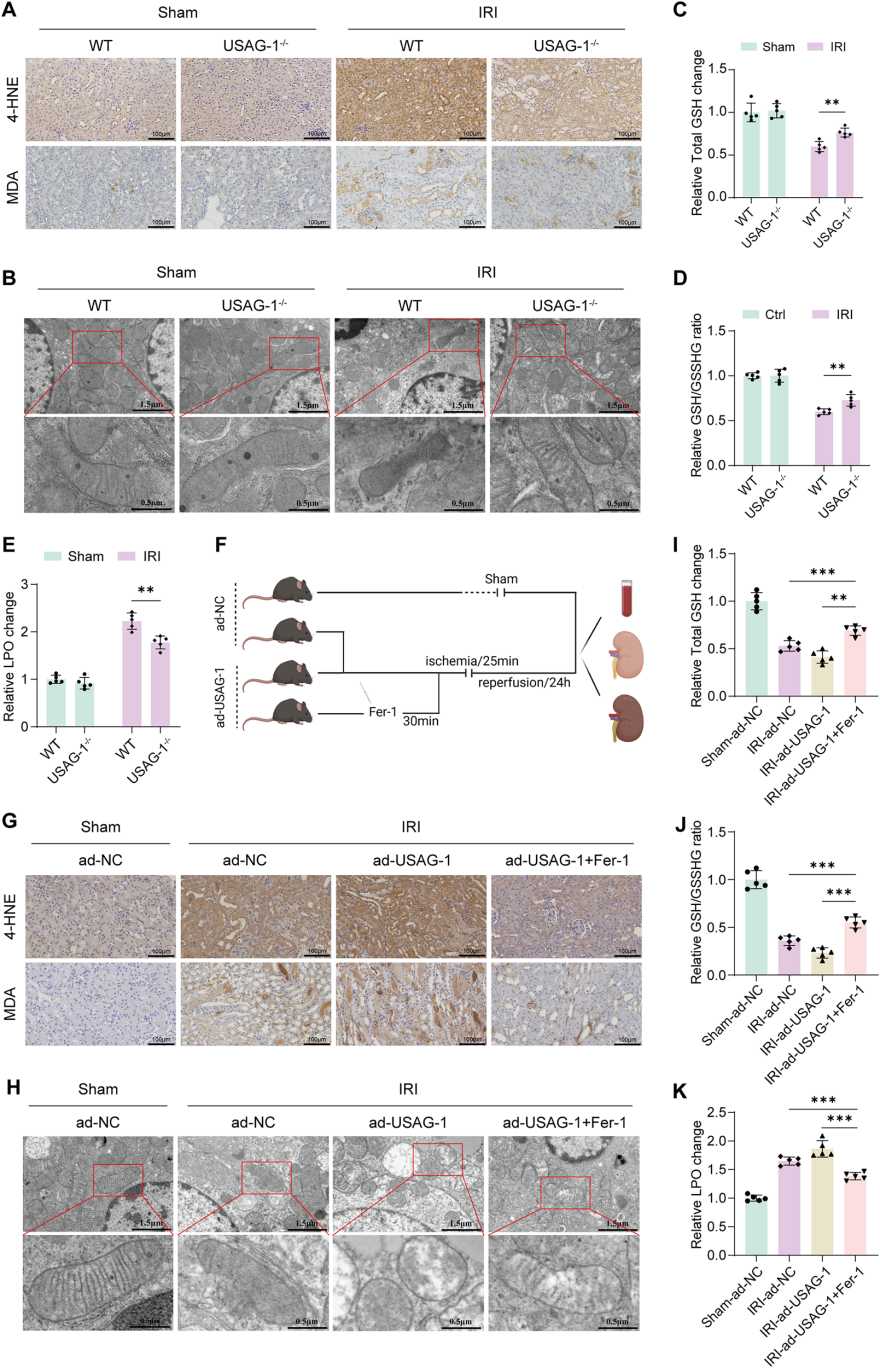
USAG-1 regulates ferroptosis in renal tubular epithelial cells during IRI. **A** Representative 4-HNE and MDA immunohistochemical staining of kidney sections from WT and USAG-1^−/−^ mice subjected to sham or IRI treatment (scale bar, 50 μm; *n* = 5). **B** Transmission electron microscopy (TEM) analysis of mitochondrial morphology in proximal tubular cells from WT and USAG-1^−/−^ mice subjected to sham or IRI treatment. **C** Measurement of total glutathione (GSH) levels in renal tissues from WT and USAG-1^−/−^ mice subjected to sham or IRI treatment (*n* = 5). **D** Measurement of the GSH/GSSG ratio in renal tissues from WT and USAG-1^−/−^ mice subjected to sham or IRI treatment (*n* = 5). **E** Measurement of LPO levels in renal tissues from WT and USAG-1^−/−^ mice subjected to sham or IRI treatment (*n* = 5). **F** Schematic diagram of the experimental groups and procedures. **G** Representative 4-HNE and MDA immunohistochemical staining of renal tissues from ad-NC, ad-USAG-1, and ad-USAG-1 + Fer-1 mice subjected to IRI treatment (scale bar, 50 μm; *n* = 5). **H** TEM analysis of mitochondrial morphology in proximal tubular cells from ad-NC, ad-USAG-1, and ad-USAG-1 + Fer-1 mice subjected to IRI treatment. **I** Measurement of total GSH levels in renal tissues from ad-NC, ad-USAG-1, and ad-USAG-1 + Fer-1 mice subjected to IRI treatment (*n* = 5). **J** Measurement of the GSH/GSSG ratio in renal tissues from ad-NC, ad-USAG-1, and ad-USAG-1 + Fer-1 mice subjected to IRI treatment (*n* = 5). **K** Measurement of LPO levels in renal tissues from ad-NC, ad-USAG-1, and ad-USAG-1 + Fer-1 mice subjected to IRI treatment (*n* = 5). ns: *p* > 0.05; *p < 0.05; **p < 0.01; ***p < 0.001.

To validate these findings in vivo, we examined the alterations of the ferroptosis pathway in the renal IRI model of USAG-1^−/−^ mice. Compared with wild-type controls, USAG-1^−/−^ mice presented significant suppression of ferroptosis-related features following IRI, including reduced levels of lipid peroxidation products (4-hydroxynonenal [4-HNE] and MDA) and diminished lipid peroxidation (LPO) accumulation (Fig. 4A, E). Moreover, total glutathione (GSH) content and the GSH/GSSG ratio were significantly increased in the kidneys of USAG-1^−/−^ mice (Fig. 4C, D). Since mitochondrial morphological alterations are hallmarks of ferroptosis, transmission electron microscopy was performed and revealed that USAG-1 deletion alleviated IRI-induced mitochondrial damage, evidenced by reduced mitochondrial shrinkage, outer membrane rupture, and cristae collapse (Fig. 4B). These results suggest that the deleterious effect of USAG-1 in renal IRI is mediated through the promotion of ferroptosis and that genetic ablation of USAG-1 mitigates ferroptotic injury.

To further clarify the role of ferroptosis in USAG-1-mediated IRI progression, we pretreated USAG-1-overexpressing mice with the ferroptosis inhibitor Fer-1 prior to IRI induction (Fig. 4F). Renal function assessments revealed that USAG-1 overexpression significantly exacerbated kidney injury, as indicated by elevated Scr and BUN levels, while pretreatment with Fer-1 effectively reversed these effects (Supplementary Fig. S5A, B). Histological evaluation supported these findings, with HE and PAS staining showing that Fer-1 markedly alleviated tubular epithelial cell vacuolization and cast formation induced by USAG-1 overexpression (Supplementary Fig. S5C, D). In addition, the protein expression levels of KIM-1 and NGAL significantly decreased following Fer-1 treatment (Supplementary Fig. S5E–G). Ferroptosis-related parameters also improved with Fer-1 pretreatment, including decreased levels of 4-HNE, MDA, and LPO (Fig. 4G, K), along with increased total GSH content and GSH/GSSG ratio (Fig. 4I, J). Ultrastructural analysis confirmed that Fer-1 pretreatment reversed the mitochondrial damage associated with USAG-1 overexpression (Fig. 4H). Collectively, these data indicate that Fer-1 mitigates USAG-1–driven exacerbation of IRI and ferroptosis, further supporting ferroptosis as a key downstream mechanism by which USAG-1 aggravates kidney injury.

To evaluate whether the protective effects of USAG-1 deletion extend beyond the IRI model, we subjected both wild-type (WT) and USAG-1^−/−^ mice to folic acid (FA)-induced AKI (Supplementary Fig. S6A). In the absence of FA treatment, there were no significant differences between WT and USAG-1^−/−^ mice in terms of renal function (Scr and BUN levels; Supplementary Fig. S6B, C) or histopathological scores (Supplementary Fig. S6D, E). However, following FA administration, USAG-1^−/−^ mice presented significantly improved renal function (lower Scr and BUN levels; Supplementary Fig. S6B, C) and milder renal injury, as evidenced by HE and PAS staining (Supplementary Fig. S6D, E). Consistently, expression levels of KIM-1 and NGAL were significantly lower in USAG-1^−/−^ mice compared to WT controls (Supplementary Fig. S6F–H). Evaluation of ferroptosis-related features further revealed that USAG-1 deficiency significantly suppressed FA-induced ferroptosis, as evidenced by decreased 4-HNE, MDA, and LPO levels (Supplementary Fig. S6D, I), along with elevated GSH levels and an increased GSH/GSSG ratio (Supplementary Fig. S6J, K). Transmission electron microscopy confirmed that USAG-1 knockout alleviated mitochondrial damage typically observed in FA-induced AKI (Supplementary Fig. S6L). In summary, these findings demonstrate that USAG-1 deficiency confers protection against both IRI- and FA-induced AKI, primarily by attenuating ferroptosis.

### USAG-1 suppresses GPX4 protein expression

**Fig. 5:**
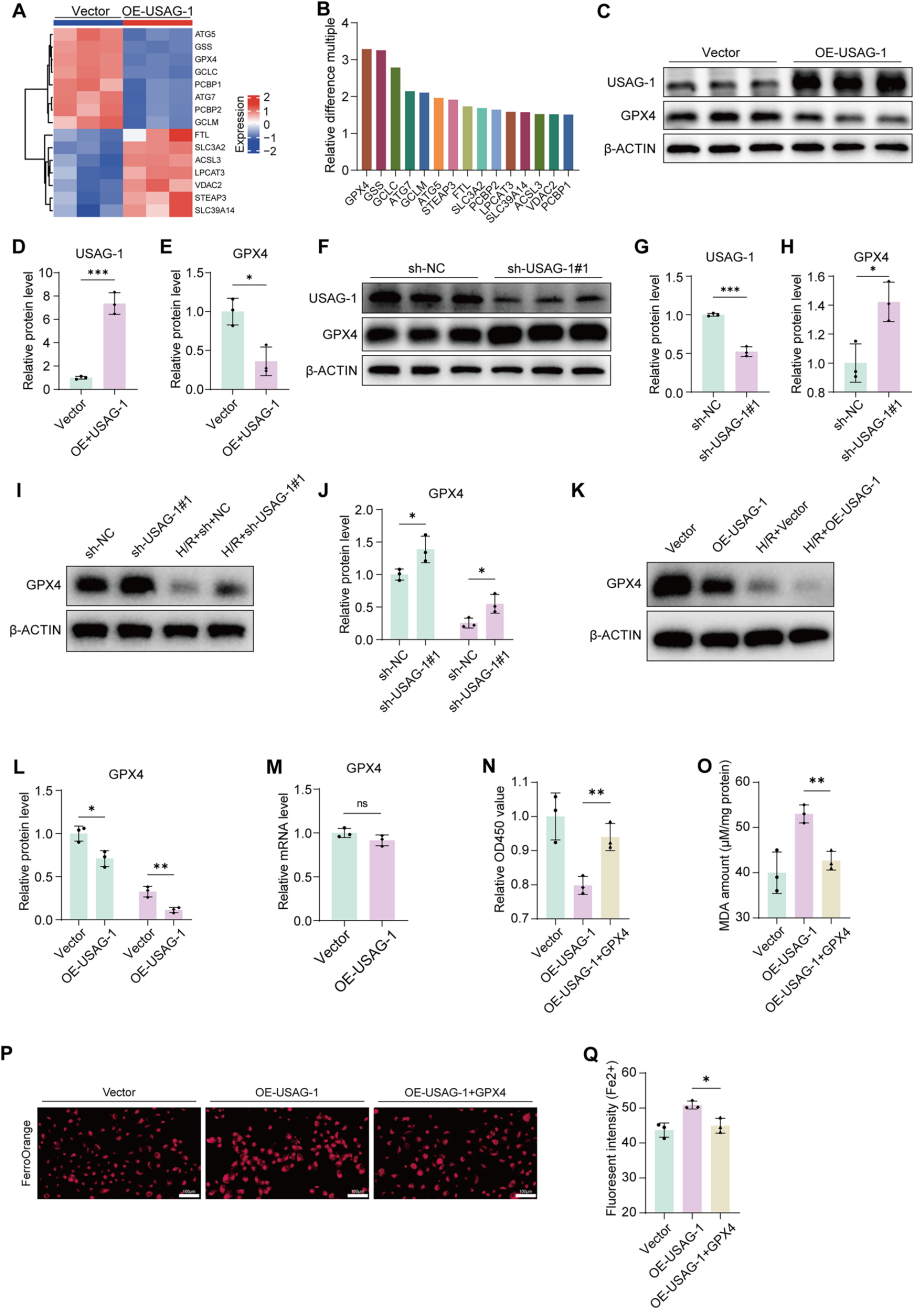
USAG-1 suppresses GPX4 protein expression. **A**, **B** Heatmap showing differentially expressed proteins enriched in KEGG pathways and the results of the fold change analysis of the indicated proteins. **C**–**E** Western blot analysis and quantification of GPX4 expression in HK-2 cells following transient USAG-1 overexpression (*n* = 3). **F**–**H** Western blot analysis and quantification of GPX4 expression in HK-2 cells following stable USAG-1 knockdown (*n* = 3). **I**, **J** Western blot analysis of GPX4 expression in HK-2 cells with stable USAG-1 knockdown under Control and H/R conditions. (*n* = 3). **K**, **L** Western blot analysis of GPX4 expression in HK-2 cells with transient USAG-1 overexpression under Control and H/R conditions (*n* = 3). **M** RT‒qPCR analysis of GPX4 mRNA levels following transient USAG-1 overexpression in HK-2 cells. **N** Cell viability of USAG-1-overexpressing HK-2 cells with concomitant GPX4 overexpression. **O** MDA levels in USAG-1-overexpressing HK-2 cells with concomitant GPX4 overexpression. **P**, **Q** Fe^2+^ levels in USAG-1-overexpressing HK-2 cells with concomitant GPX4 overexpression. ns: *p* > 0.05; *p < 0.05; **p < 0.01; ***p < 0.001.

To elucidate the molecular mechanisms by which USAG-1 regulates ferroptosis, we identified proteins enriched in the ferroptosis signaling pathway from proteomic data. A heatmap was generated to illustrate the changes among these proteins (Fig. 5A), which revealed glutathione peroxidase 4 (GPX4) as the most significantly altered target (Fig. 5B). Subsequent in vitro experiments validated the proteomic results: USAG-1 overexpression led to a marked reduction in GPX4 protein levels, whereas USAG-1 knockdown significantly increased its expression (Fig. 5C–H). We further examined the impact of USAG-1 on GPX4 expression in the H/R model. USAG-1 knockdown partially restored GPX4 levels suppressed by H/R injury, while USAG-1 overexpression further decreased GPX4 expression (Fig. 5I–L). Interestingly, although USAG-1 overexpression decreased GPX4 protein levels, RT‒qPCR analysis revealed no significant change in GPX4 mRNA levels, suggesting that USAG-1 regulates GPX4 expression at the post-transcriptional level (Fig. 5M). To determine whether GPX4 mediates USAG-1–induced ferroptosis, we co-overexpressed GPX4 in USAG-1–overexpressing HK-2 cells. Notably, GPX4 overexpression effectively restored cell viability and reversed the USAG-1–induced increases in MDA and Fe²⁺ levels (Fig. 5N–Q). Collectively, these findings suggest that USAG-1 may regulate ferroptosis by suppressing GPX4 protein expression through post-transcriptional regulation.

### HSPA5 is a direct binding partner of USAG-1 and inhibits GPX4 protein degradation

As a secreted protein, it remains unclear whether USAG-1 inhibits GPX4 through intracellular or extracellular mechanisms. Using confocal microscopy, we observed that USAG-1 was predominantly localized in the cytoplasm of HK-2 cells, with minimal nuclear presence (Supplementary Fig. S7A). Given that the N-terminal signal peptide of USAG-1 mediates its secretion, we constructed a truncated form lacking the signal peptide (USAG-1-ΔSP) and transfected HK-2 cells with either wild-type USAG-1 (USAG-1-WT) or USAG-1-ΔSP. Western blot analysis showed that USAG-1-ΔSP significantly suppressed GPX4 protein expression, similar to the full-length form (Supplementary Fig. S7B), suggesting that USAG-1 regulates GPX4 predominantly through intracellular, cytoplasmic mechanisms.

**Fig. 6:**
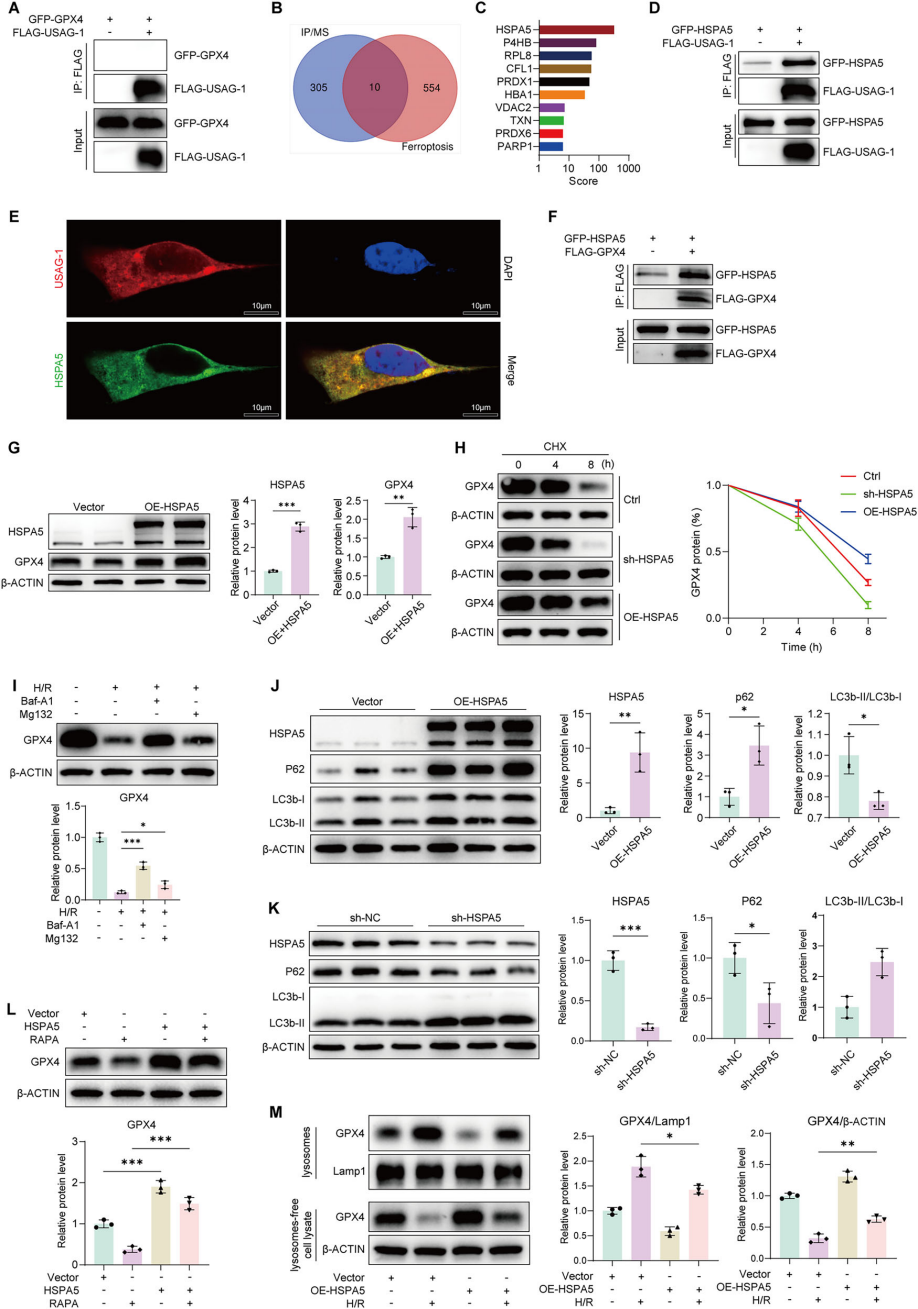
HSPA5 is a direct binding partner of USAG-1 and inhibits GPX4 protein degradation. **A** Co-IP analysis of the interaction between USAG-1 and GPX4. **B**, **C** Venn diagram and binding score analysis of proteins identified by IP/MS that interact with USAG-1 and are associated with ferroptosis. **D** Co-IP analysis of the interaction between USAG-1 and HSPA5. **E** Representative confocal immunofluorescence images showing the co-localization of USAG-1 and HSPA5 in HK-2 cells. **F** Co-IP analysis of the interaction between HSPA5 and GPX4. **G** Western blot analysis and quantification of GPX4 protein expression following HSPA5 overexpression in HK-2 cells (*n* = 3). **H** Western blot analysis and quantification of GPX4 protein levels at different time points after cycloheximide (CHX) treatment in HK-2 cells with HSPA5 overexpression or knockdown (*n* = 3). **I** Western blot analysis and quantification of GPX4 protein levels in HK-2 cells pretreated with bafilomycin A1 (Baf-A1) or MG132 before H/R exposure (*n* = 3). **J** Western blot analysis and quantification of autophagy-related markers in HK-2 cells following HSPA5 overexpression (*n* = 3). **K** Western blot analysis and quantification of autophagy-related markers in HK-2 cells following HSPA5 knockdown (*n* = 3). **L** Western blot analysis and quantification of GPX4 protein levels after rapamycin treatment in HK-2 cells overexpressing HSPA5 (*n* = 3). **M** HK-2 cells overexpressing HSPA5 were subjected to H/R injury. Lysosomal fractions and whole-cell lysates depleted of lysosomes were collected, and GPX4 protein levels were assessed by Western blotting. LAMP1 and β-actin served as loading controls for lysosomal and cytoplasmic fractions, respectively. ns: *p* > 0.05; *p < 0.05; **p < 0.01; ***p < 0.001.

Building on the earlier finding that USAG-1 regulates GPX4 at the protein rather than mRNA level, co-immunoprecipitation (Co-IP) assays revealed no direct interaction between USAG-1 and GPX4 (Fig. 6A), indicating an indirect mode of regulation. To identify potential mediators, we performed immunoprecipitation followed by mass spectrometry (IP/MS) to screen for USAG-1–interacting proteins. Cross-referencing the resulting candidates with the ferroptosis-related gene set from FerrDb identified 10 overlapping molecules, with HSPA5 exhibiting the highest interaction score (Fig. 6B, C). Consistent with the IP/MS data, both Co-IP and immunofluorescence co-localization confirmed a physical interaction between USAG-1 and HSPA5 (Fig. 6D, E).

HSPA5 (also known as GRP78 or BiP), a member of the HSP70 family, has been reported to bind GPX4 under stress conditions to prevent its degradation, thereby enhancing cellular resistance to ferroptosis [28]. Subsequent experiments demonstrated that HSPA5 directly interacts with GPX4 and enhances its protein expression (Fig. 6F, G). Moreover, cycloheximide (CHX) chase assays revealed that HSPA5 stabilizes GPX4 by preventing its degradation, consistent with previous studies (Fig. 6H). These findings led us to hypothesize that USAG-1 regulates GPX4 indirectly via HSPA5. Although HSPA5 is known to preserve GPX4 stability, the precise degradation pathway involved remains incompletely defined [29]. To investigate the dominant mechanism under H/R stress, HK-2 cells were pretreated with either an autophagy inhibitor or a ubiquitination inhibitor prior to H/R exposure. Western blot analysis revealed that autophagy inhibition but not ubiquitination blockade significantly attenuated the H/R-induced downregulation of GPX4 protein expression, suggesting that autophagy is the predominant pathway involved (Fig. 6I). To further explore the role of HSPA5 in this process, we examined the expression of autophagy-related markers, including p62 and the LC3B-II/LC3B-I ratio, following HSPA5 overexpression or knockdown. HSPA5 overexpression suppressed autophagy, as evidenced by increased p62 levels and a reduced LC3B-II/LC3B-I ratio, whereas HSPA5 knockdown had the opposite effect (Fig. 6J, K). HSPA5 overexpression also reversed the GPX4-lowering effect of the autophagy activator rapamycin (Fig. 6L). Moreover, we further investigated the impact of HSPA5 overexpression on GPX4 levels in lysosomal and non-lysosomal compartments of HK-2 cells during hypoxia-reoxygenation–induced injury (Fig. 6M), further supporting the notion that HSPA5 stabilizes GPX4 by inhibiting its autophagic degradation under stress conditions.

### USAG-1 competes with GPX4 for HSPA5 binding, promoting GPX4 degradation

**Fig. 7:**
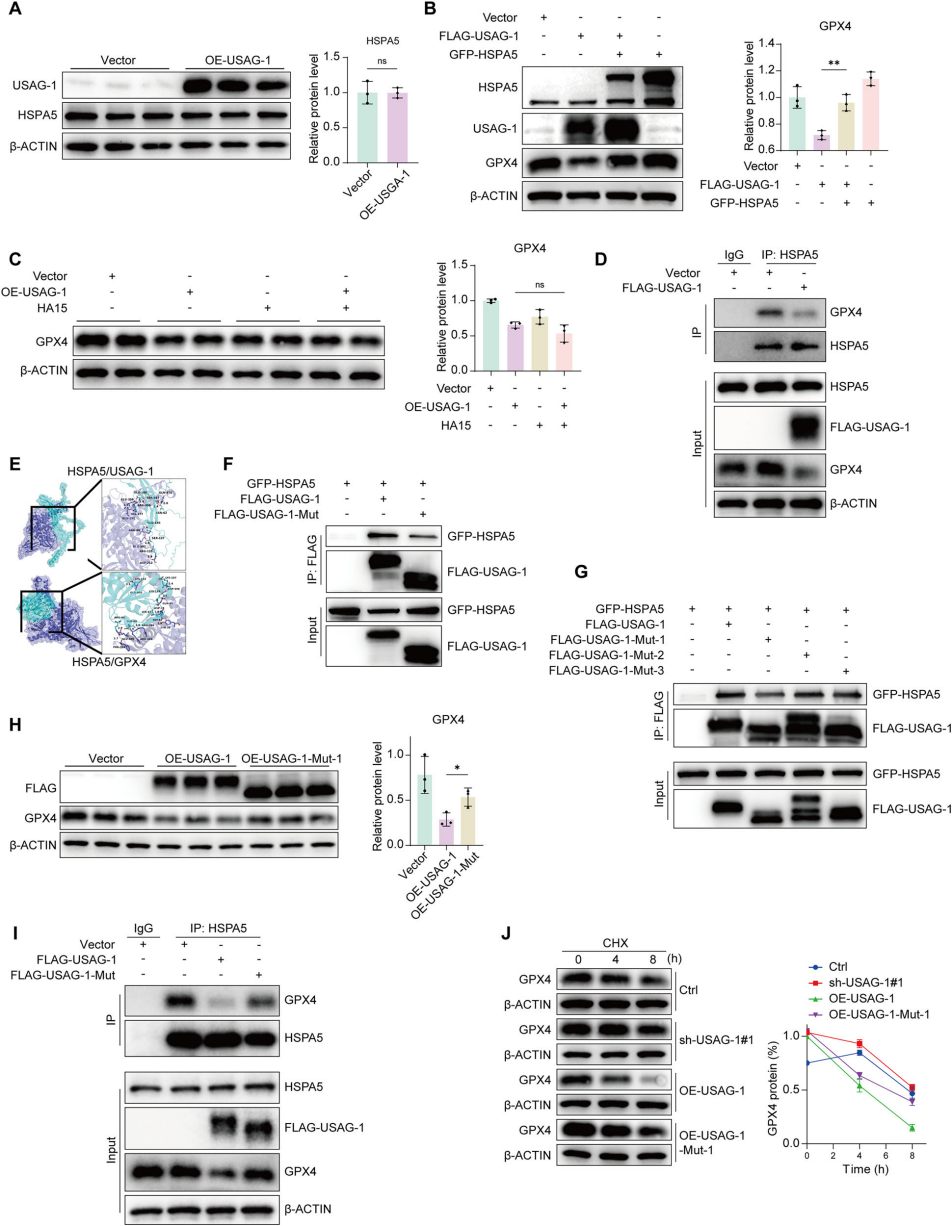
USAG-1 competes with GPX4 for HSPA5 binding, promoting GPX4 degradation. **A** Western blot analysis and quantification of HSPA5 and GPX4 protein levels following transient USAG-1 overexpression in HK-2 cells (*n* = 3). **B** Western blot analysis and quantification of GPX4 protein levels following the co-overexpression of USAG-1 and HSPA5 in HK-2 cells (*n* = 3). **C** Western blot analysis and quantification of GPX4 protein levels after USAG-1 overexpression combined with HSPA5 inhibition by HA15 in HK-2 cells (*n* = 3). **D** Co-IP assays were used to examine the interaction between HSPA5 and GPX4 after USAG-1 overexpression. **E** 3D structural models of the USAG-1, GPX4, and HSPA5 proteins obtained from the Protein Data Bank (https://www.rcsb.org/), with molecular docking and visualization performed using PyMOL software. **F** Co-IP analysis of the interaction between truncated mutants of USAG-1 (USAG-1-Mut) and HSPA5. **G** Co-IP analysis of the interactions between HSPA5 and the indicated USAG-1 truncation mutants (USAG-1, USAG-1-Mut-1, USAG-1-Mut-2, and USAG-1-Mut-3). **H** Western blot analysis of GPX4 protein expression following the overexpression of USAG-1 or USAG-1-Mut-1 in HK-2 cells. **I** Co-IP analysis of the effects of overexpressing USAG-1 or USAG-1-Mut-1 on the interaction between HSPA5 and GPX4. **J** Western blot analysis and quantification of GPX4 protein levels at different time points after cycloheximide (CHX) treatment in HK-2 cells with USAG-1 knockdown, USAG-1 overexpression, or USAG-1-Mut-1 overexpression. ns: *p* > 0.05; *p < 0.05; **p < 0.01; ***p < 0.001.

To further explore how USAG-1 modulates GPX4 protein stability, we first assessed whether USAG-1 affects HSPA5 expression. Western blot analysis revealed that USAG-1 overexpression had no significant effect on the HSPA5 protein level (Fig. 7A). However, co-overexpression of HSPA5 and USAG-1 attenuated the suppressive effect of USAG-1 on GPX4 expression (Fig. 7B). Conversely, the pharmacological inhibition of HSPA5 using HA15 abolished the ability of USAG-1 overexpression to further downregulate GPX4 (Fig. 7C), indicating that HSPA5 plays a critical role in mediating USAG-1-induced GPX4 downregulation, although not by altering HSPA5 expression itself. Given that both USAG-1 and GPX4 were shown to interact directly with HSPA5—but not with each other—we hypothesized that USAG-1 disrupts the HSPA5–GPX4 interaction through competitive binding. Supporting this hypothesis, Co-IP assays demonstrated that USAG-1 overexpression significantly reduced the binding of HSPA5 to GPX4 (Fig. 7D). In silico molecular docking further predicted overlapping HSPA5-binding interfaces for both USAG-1 and GPX4 (Fig. 7E). To validate this mechanism experimentally, we constructed a USAG-1 mutant (USAG-1-Mut) lacking all the predicted HSPA5-interacting domains. Co-IP assays showed that USAG-1-Mut had a markedly reduced capacity to bind HSPA5 compared to wild-type USAG-1 (Fig. 7F), confirming the validity of the mutation and supporting the notion that USAG-1 interacts with HSPA5.

To delineate the specific HSPA5-binding domains on USAG-1 responsible for this disruption, we generated three truncation mutants (USAG-1-Mut-1, -Mut-2, and -Mut-3), each lacking a different predicted interaction site (Supplementary Fig. S8A). Co-IP analysis revealed that USAG-1-Mut-1, which lacks the domain overlapping with GPX4’s binding site on HSPA5, exhibited the greatest reduction in HSPA5 interaction (Fig. 7G). Functionally, overexpression of USAG-1-Mut-1 restored GPX4 protein levels, effectively reversing the suppressive effect seen with wild-type USAG-1 (Fig. 7H). Moreover, USAG-1-Mut-1 failed to disrupt the HSPA5–GPX4 complex, as confirmed by Co-IP (Fig. 7I), providing strong evidence for a competitive binding mechanism. Finally, cycloheximide chase assays were performed to evaluate the impact of USAG-1 on GPX4 protein stability. Knockdown of USAG-1 significantly delayed GPX4 degradation, whereas overexpression of wild-type USAG-1 accelerated it. In contrast, USAG-1-Mut-1 overexpression had minimal effect on GPX4 degradation kinetics (Fig. 7J), further corroborating the role of the HSPA5-binding domain in USAG-1-mediated GPX4 destabilization.

**Fig. 8:**
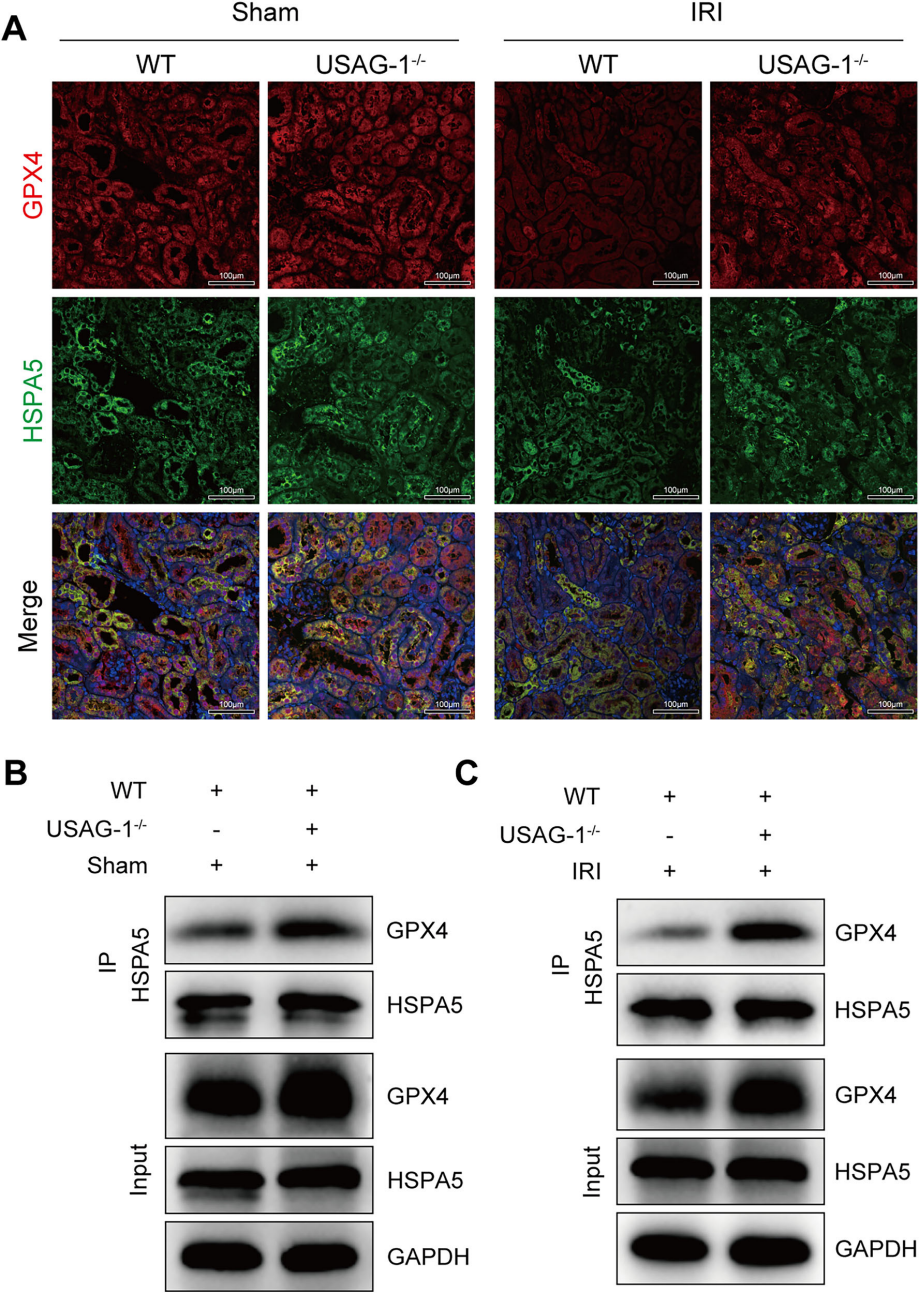
USAG-1 deficiency enhances the interaction between HSPA5 and GPX4. **A** Representative IF staining of GPX4 (red), HSPA5 (green), and DAPI (blue) in kidney sections from WT and USAG-1^−/−^ mice subjected to sham or IRI treatment (scale bar: 100 μm). **B**, **C** Co-IP analysis of kidney tissues from different groups of mice was performed to investigate the effects of USAG-1 deficiency on the interaction between HSPA5 and GPX4.

This competitive interaction mechanism was further validated in vivo. Immunofluorescence staining in both sham and IRI groups revealed markedly enhanced co-localization of HSPA5 and GPX4 in the kidneys of USAG-1^−/−^ mice compared to wild-type controls (Fig. 8A). Consistent with this, Co-IP analysis demonstrated a significantly stronger HSPA5–GPX4 interaction in USAG-1-deficient kidneys (Fig. 8B, C), indicating that loss of USAG-1 preserves the HSPA5–GPX4 complex under both physiological and stress conditions. Together, these findings demonstrate that USAG-1 promotes GPX4 degradation by competitively binding to HSPA5 at the GPX4 interaction site, thereby disrupting the protective HSPA5–GPX4 complex and sensitizing renal tubular cells to ferroptosis.

## Discussion

This study identified a novel role of USAG-1 in renal IRI and elucidated its underlying mechanism. Under ischemic and hypoxic conditions, USAG-1 protein levels were markedly elevated in both the murine IRI model and transplant biopsies from DCD donor kidneys, and this expression positively correlated with ischemic severity. These findings suggest that USAG-1 is induced by ischemic stress and contributes to the pathophysiology of IRI. Functional experiments further showed that genetic deletion of USAG-1 attenuated acute tubular injury and renal dysfunction after IRI, whereas USAG-1 overexpression aggravated renal injury. Together, these findings support a deleterious role for USAG-1 in renal IRI and provide new insight into the molecular regulation of ischemic kidney injury.

During IRI, tissues are exposed to hypoxia, reoxygenation, inflammatory infiltration, and oxidative stress, all of which may contribute to USAG-1 induction through complex signaling networks [30]. Previous studies suggest that USAG-1 is dynamically regulated according to injury stage; in a folic acid-induced kidney injury model, it decreases during the acute phase but increases during the repair phase [24]. In contrast, in our IRI model, USAG-1 increased during ischemia/hypoxia but declined after reperfusion. These findings suggest that USAG-1 responds differently depending on injury type and severity. Furthermore, prolonged ischemic time was correlated with both aggravated tubular injury and progressively increased USAG-1 protein levels. These findings imply that ischemic stress induces an early USAG-1 response; however, this response appears to be detrimental, potentially exacerbating tissue damage through specific pathways such as ferroptosis. Although USAG-1 exhibits a relatively narrow temporal window, this pattern may itself be pathophysiologically informative. This temporal profile suggests a critical early window during which USAG-1 may initiate downstream signaling and contribute to subsequent ferroptotic injury. Accordingly, the translational value of USAG-1 may lie less in delayed postoperative monitoring and more in peri-reperfusion risk assessment and pre-emptive intervention. In kidney transplantation, this supports evaluating USAG-1 in implantation-time or time-zero biopsies and suggests that USAG-1-targeted strategies may be best explored during graft procurement, preservation, machine perfusion, or the immediate peri-reperfusion phase. At the same time, the upstream mechanisms responsible for ischemia-induced USAG-1 upregulation remain incompletely understood and warrant further investigation.

Our study is the first to establish a pathogenic role for USAG-1 in ferroptosis during renal IRI. Renal tubular epithelial cells are highly susceptible to ferroptosis in IRI, largely because iron accumulation and ROS drive lipid peroxidation and membrane damage [31]. In the present study, USAG-1 deletion reduced lipid peroxidation and ferroptosis-associated markers in renal tissues after IRI, whereas USAG-1 overexpression aggravated these changes. These findings suggest that elevated USAG-1 increases tubular susceptibility to ferroptosis and thereby worsens tissue injury. Given that ferroptosis has emerged as a promising therapeutic target in AKI and IRI [12], targeting the USAG-1-mediated ferroptotic pathway may represent a novel strategy to mitigate IRI-associated renal damage.

Previous studies have mainly characterized USAG-1 as a negative regulator of renal repair, with its detrimental effects largely attributed to antagonism of BMP7-mediated regenerative signaling. Yanagita and colleagues identified USAG-1 as the most abundant BMP antagonist in the adult kidney across murine models of kidney injury. They further showed that USAG-1 deficiency preserved renal function in cisplatin-induced AKI and UUO-induced chronic kidney injury [19, 21]. Together, these studies indicate that in chronic injury or fibrosis, USAG-1 limits tissue repair and that its absence is protective. Our findings are broadly consistent with these observations, as USAG-1 also acts as a pro-injury factor in acute ischemic injury, and its deletion mitigates renal damage. However, our study expands the mechanistic understanding of USAG-1 beyond the classical BMP7/Smad axis. Specifically, we identified a pathogenic mechanism by which USAG-1 exacerbates acute tubular injury through ferroptosis. Although USAG-1 is classically recognized as a BMP7 antagonist, and BMP7/Smad signaling has been most extensively studied in kidney repair and fibrosis, the protective effects of this pathway in AKI and IRI have also been reported [23, 32, 33]. In contrast to the classical BMP7/Smad axis, HSPA5 is more directly linked to endoplasmic reticulum stress, proteostasis, and acute stress adaptation. At present, direct evidence linking BMP7 to HSPA5 in renal IRI remains limited. Thus, our findings do not support a simple linear BMP7–HSPA5 pathway. Rather, they suggest that USAG-1 may contribute to kidney injury through both its classical modulation of BMP7 signaling and the newly identified HSPA5/GPX4-dependent ferroptosis mechanism. Whether these two pathways function independently, sequentially, or in a coordinated manner during renal IRI remains to be clarified. Together, these data suggest that USAG-1 exerts multifaceted effects in kidney injury, limiting BMP-driven regeneration in chronic settings while directly influencing tubular cell survival through ferroptosis in the acute phase.

A central aspect of this study is the regulation of ferroptosis, particularly through GPX4, a key suppressor of ferroptotic cell death. GPX4 inhibits lipid peroxidation by reducing phospholipid hydroperoxides and serves as a critical safeguard of cellular redox homeostasis [34, 35]. Its importance in renal ischemic injury is underscored by the finding that conditional GPX4 deletion causes extensive tubular epithelial cell death and severe AKI in mice [12]. Consequently, factors impairing GPX4 function are likely to exacerbate IRI-induced cell death. In this context, our study identifies a mechanism by which USAG-1 indirectly compromises GPX4 protein stability. Unlike mechanisms that directly inhibit GPX4 activity or promote its autophagic degradation [36], USAG-1 does not appear to alter GPX4 enzymatic activity itself. Instead, USAG-1 interferes with HSPA5, a critical chaperone required for GPX4 stability, thereby reducing intracellular GPX4 levels. This represents a novel mode of modulating ferroptosis, highlighting that in addition to enzymatic regulation and metabolic pathways, protein‒protein interactions and homeostatic maintenance play pivotal roles in ferroptotic control. Compared with systemic GPX4 modulation, which may carry immunologic, signaling, metabolic, or oncogenic risks, targeting USAG-1 may offer a more cell-specific approach in tubular epithelial cells [37]. This selectivity may provide therapeutic advantages in managing renal IRI while minimizing systemic side effects.

**Fig. 9:**
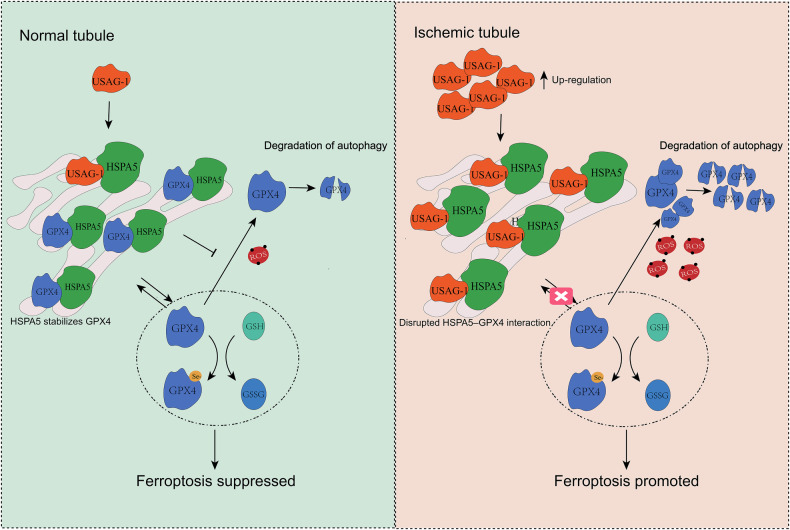
Schematic illustration of the proposed role of USAG-1 in ferroptosis during renal IRI. Under normal conditions, HSPA5 binds to and stabilizes GPX4, thereby limiting ferroptosis. Under ischemic and hypoxic conditions, USAG-1 is markedly upregulated and competitively interferes with the interaction between HSPA5 and GPX4, thereby facilitating GPX4 degradation, promoting ferroptosis, and aggravating tubular cell injury.

As a key component of the ER stress response, HSPA5 binds unfolded or misfolded proteins, thereby preventing their aggregation or degradation and promoting cell survival under stress [38]. Recent studies have shown that upregulated HSPA5 directly binds and stabilizes GPX4, thereby protecting it from degradation and suppressing ferroptosis [28]. In this context, HSPA5 functions as a stabilizing partner of GPX4 through complex formation. In our study, USAG-1 directly interacted with HSPA5, and molecular docking predicted overlapping HSPA5-binding sites for USAG-1 and GPX4. These findings suggest that USAG-1 may competitively bind HSPA5, disrupt HSPA5-mediated GPX4 stabilization, and thereby promote GPX4 degradation. Consistent with this mechanism, USAG-1 overexpression reduced GPX4 protein levels and increased lipid peroxidation and ferroptosis markers. Conversely, USAG-1 knockout preserved HSPA5 function, stabilized GPX4, and consequently suppressed ferroptosis. To our knowledge, no previous studies have reported a connection between USAG-1 and HSPA5. Our findings identify USAG-1 as a novel regulatory factor within the HSPA5–GPX4 axis. This proposed mechanism highlights a previously unrecognized mode of regulation in which extracellular proteins such as USAG-1 modulate intracellular chaperone activity to influence cell fate (Fig. 9). This pathway provides new insight into the control of cell death in IRI and may inform therapeutic strategies aimed at preserving HSPA5 function or preventing the USAG-1–HSPA5 interaction.

As a secretory protein, USAG-1 is synthesized and trafficked through the endoplasmic reticulum (ER)-Golgi pathway for extracellular release. Our study revealed prominent co-localization of USAG-1 with HSPA5 in the cytoplasmic compartment. Moreover, a signal peptide-deficient USAG-1 mutant (ΔSP-USAG-1), which is retained intracellularly due to impaired secretion, still disrupted the HSPA5–GPX4 regulatory axis. These findings strongly suggest that USAG-1 engages HSPA5 within the ER lumen prior to secretion, thereby competitively impairing the chaperone activity of HSPA5 toward GPX4 stabilization. Future studies are warranted to map the precise binding interface and critical amino acid residues governing the USAG-1/HSPA5 interaction. Defining these molecular determinants will refine our understanding of how USAG-1 disrupts the HSPA5–GPX4 cytoprotective axis and may facilitate the design of therapeutic agents targeting this interaction.

### Clinical implications

These findings have important clinical implications for kidney transplantation. First, USAG-1 may serve as a promising biomarker for DGF risk stratification. Current DGF risk assessment relies mainly on donor characteristics and graft quality metrics. However, DGF remains common even with current donor selection and graft assessment strategies [39]. Our data suggest that quantifying USAG-1 expression in donor kidneys may refine early risk stratification for DGF severity and duration. Second, USAG-1 may represent a therapeutic target for limiting ferroptosis-driven IRI. USAG-1-neutralizing antibodies or small-molecule antagonists, delivered systemically or through ex vivo graft perfusion, may mitigate ferroptosis-driven IRI, reduce DGF incidence, and improve early graft function. Such an approach would address the unmet need for therapies targeting the molecular basis of IRI rather than its downstream consequences.

## Methods

### Antibodies and plasmids

The following antibodies were purchased from commercial vendors: rabbit anti-USAG-1 (ab99340, Abcam); rabbit anti-GPX4 (CY6959, Abways); rabbit anti-HSPA5 (66574-1-Ig, Proteintech); rabbit anti-NGAL (26991-1-AP, Proteintech); rabbit anti-Kim-1 (30948-1-AP, Proteintech); rabbit anti-4-HNE (ab48506, Abcam); rabbit anti-MDA (ab243066, Abcam); rabbit anti-p62 (ab109012, Abcam); rabbit anti-LC3b (ab192890, Abcam); mouse anti-Actin (66009-1-Ig, Proteintech); rabbit anti-Flag (20543-1-AP, Proteintech); rabbit anti-GFP (AB0045, Abways); rabbit anti-NKCC2 (18970-1-AP, Proteintech); rabbit anti-AQP2 (29386-1-AP, Proteintech); rabbit anti-Calbindin-D28K (14479-1-AP, Proteintech); anti-rabbit IgG, HRP-linked antibody (7074, Cell Signaling Technology); anti-mouse IgG, HRP-linked antibody (7076, Cell Signaling Technology); CoraLite488-conjugated goat anti-rabbit IgG (SA00013-2, Proteintech); CoraLite594-conjugated Goat Anti-Rabbit IgG (SA00013-4, Proteintech); CoraLite488-conjugated Goat Anti-Mouse IgG (SA00013-1, Proteintech); CoraLite594-conjugated Goat Anti-Mouse IgG (SA00013-3, Proteintech).

The empty plasmid vector, plasmids encoding wild-type USAG-1 (Flag-USAG-1 and GFP-USAG-1), truncated USAG-1 mutants (USAG-1-Mut, USAG-1-Mut-1, USAG-1-Mut-2, USAG-1-Mut-3, and USAG-1-△SP), plasmid encoding wild-type HSPA5 (GFP-HSPA5), and plasmids encoding wild-type GPX4 (GFP-GPX4 and Flag-GPX4) were all provided by Shanghai Genechem Co., Ltd.

### Patients

This study included 52 recipients who developed DGF after kidney transplantation from DCDs at the Department of Kidney Transplantation, The First Affiliated Hospital of Zhengzhou University, as the study group. Additionally, 52 recipients with stable graft function (ST) after kidney transplantation during the same period were randomly selected as controls. Zero-time biopsy samples of donor kidneys were obtained, and clinical history and follow-up data were collected. Written informed consent was obtained from all subjects or their legal representatives.

### Animals

Male C57BL/6 mice (6–8 weeks old) were purchased from Weitong Lihua Laboratory Animal Technology Co., Ltd. (Beijing, China). USAG-1-knockout (KO) and USAG-1^flox/flox^ (USAG-1^fl/fl^) mice were purchased from Model Organisms Center, Inc. (Shanghai, China). For validation of renal USAG-1 deletion, whole-kidney tissues from WT and USAG-1-KO mice were collected for RT-qPCR, Western blotting, and immunohistochemistry. RT-qPCR and Western blotting were performed using whole-kidney tissue samples/lysates, and immunohistochemistry was performed on kidney sections. All animals were maintained under constant humidity and temperature in standard facilities under specific pathogen-free conditions with free access to food and water. Animals were randomly assigned to the indicated experimental groups.

Recombinant adeno-associated viruses (AAV9) carrying a kidney tubule epithelial cell-specific promoter (Ksp-cadherin, KspC) were utilized to transduce renal tubular epithelial cells via tail vein injection. The AAV9 constructs included a negative control vector (AAV9-NC) and a USAG-1 overexpression vector (AAV9-USAG-1), each with a titer of 2.5×10^11^ vg. Subsequent experiments were performed 3 weeks after AAV administration to ensure stable gene expression. All the AAVs were provided by Shanghai Genechem Co., Ltd. (Shanghai, China).

All operations were carried out in accordance with the National Institutes of Health (NIH) guidelines for the Care and Use of Laboratory Animals and were approved by the Ethics Committee of The First Affiliated Hospital of Zhengzhou University (Ethics Approval No. ZZU-LAC20210521[07]).

### Animal model

#### Renal ischemia/reperfusion model

Our study examined male mice because male animals exhibited less variability in phenotype. It is unknown whether the findings are relevant for female mice. Renal IRI was induced by occluding the left renal artery with a microaneurysm clamp for 25 min, followed by 24 h of reperfusion. Immediately after left renal artery clamping, a right nephrectomy was performed to preclude compensatory renal function. Throughout the procedure, the mice were maintained on a warming pad to stabilize their core body temperature at 36.5–37.0 °C. Ferrostatin-1 (Fer-1, 5 mg/kg) was intraperitoneally injected 15 min before IRI induction in C57BL/6 mice.

#### Folic acid-induced AKI

For the FA-AKI model, male C57BL/6 mice (25–28 g, 6–8 weeks old) received a single intraperitoneal injection of 250 mg/kg FA dissolved in 0.3 M sodium bicarbonate or vehicle and were euthanized 48 h later. Blood samples were collected at the time of euthanasia, and the kidneys were perfused with saline before removal.

### Cell culture and treatment

HK-2 cells and HEK293 cells were obtained from Procell Life Science & Technology Co., Ltd. STR authentication was not performed. The cells were tested negative for mycoplasma contamination before experiments. HK-2 cells were maintained in Dulbecco’s modified Eagle’s medium/nutrient mixture F-12 (DMEM/F12, Gibco, USA), whereas HEK293 cells were maintained in DMEM basic medium (Gibco, USA), both supplemented with 10% fetal bovine serum (FBS, Clark USA) and 1% penicillin/streptomycin antibiotics (NCM, China) and incubated at 37 °C with 5% CO_2_. To establish the hypoxia-reoxygenation (HR) model, the cells were exposed to a hypoxic environment (1% O₂, 5% CO₂, and 94% N₂) in serum-free medium for 12 h. The cells were subsequently transferred to fresh complete medium under normoxic conditions (21% O₂, 5% CO₂, 74% N₂) for 8 h to initiate reoxygenation. After HR treatment, the cells were harvested for downstream analyses.

### Gene silencing and overexpression assays

For gene knockdown, lentiviral vectors encoding shRNAs were transduced into cells. The viruses encoding shRNAs were synthesized by Shanghai Genechem Co., Ltd. Two shRNA sequences targeting USAG-1 were used: sh-USAG-1#1, 5′-GGAACTGCGTTCCACCAAATA-3′; and sh-USAG-1#2, 5′-CAGTCACAACTTTGAGAGCAT-3′. The shRNA sequence for targeting HSPA5 (shHSPA5) was 5′-GGAACCATCCCGTGGCATAAA-3′. The knockdown efficiency was validated by Western blotting. At the cellular level, all overexpression experiments were performed by transient transfection of expression plasmids into the indicated cell lines.

### Data collection and bioinformatics analysis

GEO datasets (GSE43974, GSE30718, and GSE186316) were downloaded from the GEO database (http://www.ncbi.nlm.nih.gov/geo). We used the R package DESeq2 to identify differentially expressed genes. Adjusted *p* value < 0.05 and |log2-fold change| > 0 were set as the cutoff criteria. Differences in protein levels were evaluated using Student’s *t*-test. A *p* value < 0.01 indicated a statistically significant difference between the control and treated groups. GSEA was performed using the R package clusterProfiler. Genes were ranked on the basis of the log2-fold change. Gene Ontology (GO) knowledgebase (http://geneontology.org/) and KEGG pathway enrichment analyses (https://www.genome.jp/kegg/) were conducted using the clusterProfiler package in R 4.1.3. Volcano maps and heatmaps were generated using the R packages “ggplot2” and “pheatmap”, respectively.

### Renal function

Renal function was assessed by measuring Scr and BUN levels. Commercially available kits (#C013-2-1 for BUN, #C011-2-1 for Scr; Nanjing Jiancheng, China) were used in accordance with the manufacturer’s instructions.

### Histology

Renal tissues were embedded in paraffin, sectioned (4 μm thick), and stained with hematoxylin and eosin (H&E) and PAS for histopathological evaluation. Tubular injury was diagnostically defined as cytolysis, brush border loss, or intraluminal cast formation. A blinded semiquantitative scoring system (grades 0–5) was used to categorize injury severity by the percentage of affected tubules per field: 0, no damage; 1, <20%; 2, 20–40%; 3, 40–60%; 4, 60–80%; and 5, >80%. For each sample, 10 randomly selected cortical fields (200× magnification) were independently evaluated by two investigators who were blinded to the group allocation, and the final score was calculated as the mean value across all fields.

### Transmission electron microscopy (TEM)

Renal tissues from mice were fixed in glutaraldehyde and then post-fixed with 1% osmium tetroxide. The samples were dehydrated through a graded acetone series, infiltrated with embedding medium, and embedded in 812 epoxy resin, followed by polymerization at 60 °C. Ultrathin sections (~60 nm) were cut using an ultramicrotome, stained with uranyl acetate and lead citrate, and examined under a transmission electron microscope (Hitachi, Tokyo, Japan).

### RT‒qPCR

Total RNA was isolated from cell and tissue samples using a FastPure Cell/Tissue Total RNA Isolation Kit V2 (Vazyme Biotech Co., Ltd.). Subsequently, reverse transcription was performed using Hifair III 1st Strand cDNA Synthesis SuperMix for qPCR (YEASEN, China) to synthesize complementary DNA. To assess mRNA expression levels, real-time quantitative PCR was carried out using Hieff qPCR SYBR Green Master Mix (YEASEN, China). For data normalization, GAPDH mRNA expression was used as the endogenous control. The relative quantification of target gene expression was determined through comparative threshold cycle analysis using the 2^−ΔΔC^T calculation method.

### Cell counting kit-8 (CCK-8) assay

For the CCK-8 assays, 4000 cells were seeded per well in 96-well plates. Ten microliters of CCK-8 reagent (C6005, New Cell & Molecular Biotech, China) mixed with 90 μl of complete medium was added to each well, and the cells were incubated at 37 °C for 2 h. Then, the absorbance was measured at 450 nm.

### Western blot and Co-IP analyses

Total protein was extracted from cells and tissues by lysis in RIPA lysis buffer (Beyotime, China) supplemented with protease inhibitor (Beyotime, China). Protein concentrations were quantified using a bicinchoninic acid (BCA) assay kit. For Co-IP, the lysates were combined with anti-DYKDDDDK (Flag) Affinity Gel (YEASEN, China) and rotated at 4 °C for 4 h. The beads were pelleted by centrifugation, washed three times with TBS, resuspended in 2× loading buffer, and denatured at 95 °C for 5 min.

For Western blot analysis, proteins were separated by SDS‒PAGE using an Omni-Easy™ One-Step PAGE Gel Fast Preparation Kit (Epizyme, Shanghai) and transferred onto PVDF membranes using a wet transfer system. The membranes were blocked with 5% skim milk for 2 h at room temperature and then incubated with primary antibodies at 4 °C overnight. After being washed with TBST (3 × 10 min), the membranes were probed with HRP-conjugated secondary antibodies for 1 h. Following intensive washing with TBST (5 × 5 min), protein signals were visualized using an enhanced chemiluminescence reagent. All experimental reagents, including electrophoresis buffer, transfer buffer, TBST, and related solutions, were obtained from Yeasen Biotech (Shanghai).

For co-immunoprecipitation assays, HEK293 cells were transiently transfected with the indicated expression plasmids and harvested 48 h later for protein extraction and immunoprecipitation. HEK293 cells were used as an overexpression-based heterologous system for protein interaction validation because of their high transfection efficiency.

### MDA assay

The quantification of lipid peroxidation products was performed through the spectrophotometric measurement of malondialdehyde (MDA) levels using a commercial assay kit (Lipid Peroxidation MDA Assay Kit, Beyotime Biotechnology, Shanghai, China). After lysing cells in RIPA buffer (containing protease inhibitors), the lysate was centrifuged at 12,000 × *g* for 10 min at 4 °C, and the supernatant was retained. Protein quantification was initially conducted using a BCA protein assay in accordance with the manufacturer’s specifications. Then, the mixed MDA detection working solution was mixed with each protein sample, followed by heating at 100 °C for 15 min. After cooling to room temperature and centrifuging at 10,000 × *g* for 10 min, the chromogenic products were transferred to a 96-well microplate to measure the optical density at 532 nm using a microplate reader, with appropriate blank controls and an MDA standard curve.

### Immunohistochemistry

Immunohistochemical analysis was performed using standardized protocols. Paraffin-embedded tissue sections were subjected to heat-mediated antigen retrieval in sodium citrate buffer (pH 8.0) after deparaffinization through a xylene gradient and baking in a 60 °C oven (40 min). Endogenous peroxidase activity was quenched with 3% H₂O₂ (10 min, RT), followed by blocking with 3% BSA (30 min). The sections were incubated with primary antibodies at 4 °C overnight and subsequently with HRP-conjugated secondary antibodies (30 min, RT). Counterstaining with hematoxylin and dehydration through an ethanol series preceded microscopy image acquisition using a brightfield system.

### Immunofluorescence

Tissue pretreatment, including deparaffinization, antigen retrieval, and incubation with primary antibodies, was performed following protocols identical to those described for immunohistochemistry. Sections were subsequently incubated with fluorophore-conjugated secondary antibodies (1:500) under light-protected conditions for 1.5 h. Nuclei were counterstained with DAPI-containing antifade mounting medium (Beyotime, China) prior to image acquisition using a laser scanning confocal microscope (Zeiss LSM880, Germany). To better determine the localization of USAG-1 in renal tubules, USAG-1-EGFP reporter mice were used for immunofluorescence staining of kidney sections. For co-localization analysis of USAG-1 with proximal tubular markers (LTL), renal sections previously stained for USAG-1 were coincubated with fluorescein-conjugated LTL (Vector Laboratories, FL-1321) at room temperature (RT) for 1 h. The sections were mounted with DAPI-containing antifade medium (Beyotime Biotechnology, China) and imaged using confocal microscopy.

Cellular immunofluorescence staining was performed in accordance with standardized protocols. Cells cultured on glass-bottom dishes were fixed with 4% paraformaldehyde (15 min), permeabilized with 0.5% Triton X-100 (20 min), and blocked with an immunofluorescence-specific solution. Cells were incubated with primary antibodies overnight at 4 °C, followed by incubation for 1 h in the dark with fluorophore-conjugated secondary antibodies. Nuclear counterstaining was performed with Hoechst prior to imaging using a laser scanning confocal microscope (Zeiss LSM880, Germany). All washing steps between reagent changes were performed using PBS.

Relative fluorescence intensities were evaluated using ImageJ software (NIH, USA) after background subtraction and normalization to the control group.

### Lysosome isolation

Lysosomes were harvested by homogenization and sequential centrifugation with a lysosome isolation kit (BestBio, Shanghai, China), according to the manufacturer’s protocol.

### GSH and lipid peroxidation release assays

Clarified lysates from renal homogenates (12,000 × *g*, 10 min) were subjected to glutathione analysis (total/oxidized GSH ratio, Beyotime S0053) and lipid peroxidation quantification (LPO assay, Jiancheng A106) following the manufacturer’s instructions.

### FerroOrange and lipid reactive oxidation species detection

FerroOrange (Dojindo, F347, Japan) is a fluorescent probe that enables live-cell imaging of intracellular Fe^2+^. For the assay, cells were incubated with HBSS buffer containing 1 µmol/L FerroOrange for 1 h at 37 °C. After incubation, intracellular Fe^2+^ levels were visualized by fluorescence microscopy, and fluorescence intensity was quantified using ImageJ software (NIH, USA).

Lipid reactive oxidation species were assessed using the Lipid Peroxidation Probe BDP 581/591 C11 (Dojindo, L267, Japan) according to the manufacturer’s instructions. The fluorescence signal was captured by fluorescence microscopy, and the intensity was quantified using ImageJ software (NIH, USA).

### Statistics

Statistical analyses were conducted using GraphPad Prism 9.0 or SPSS 24.0. The data are expressed as mean ± standard error of the mean. The normality of the data distribution was formally tested, using the Shapiro–Wilk test when the sample size is less than 50, and the Kolmogorov–Smirnov test for sample sizes equal to or greater than 50. The Brown‒Forsythe test was used to evaluate the homogeneity of variance. For comparisons between two groups, an unpaired two-tailed Student’s *t*-test was applied when the data followed a normal distribution with equal variances. When variances were unequal, Welch’s *t*-test was used. Nonnormally distributed data were analyzed using the Mann–Whitney *U* test. For comparisons among three or more groups, one-way ANOVA followed by Bonferroni post hoc correction was employed for normally distributed data, whereas the Kruskal–Wallis test with Dunn’s multiple comparisons test was used for nonnormally distributed data. For multifactorial comparisons, two-way ANOVA followed by Bonferroni post hoc correction was applied for normally distributed datasets, whereas multiple Mann–Whitney *U* tests were used for nonparametric data. Risk factors for DGF were analyzed using binary logistic regression. ROC curve analysis was performed to evaluate the sensitivity and specificity of donor and recipient characteristics in predicting DGF. The area under the ROC curve (AUC) was calculated to assess the predictive accuracy. For the majority of experiments, we performed three biological replicates per group. This sample size was determined based on previous studies in the literature that assessed similar experimental conditions and outcomes, and was deemed sufficient for detecting meaningful differences given the nature of the experiments [40]. No formal blinding was performed during group allocation, experiment execution, or data collection. No animals, human samples, or experimental replicates were excluded from the analyses, and no formal inclusion or exclusion criteria were pre-established. A *P* value < 0.05 was considered statistically significant.

### Ethics approval

The clinical study involving clinical specimens was approved by the Institutional Review Board of the First Affiliated Hospital of Zhengzhou University in Zhengzhou, China (Ethics Approval Number: 2025-KY-0462-001; Clinical study registration number: ChiCTR2500109330), and complied with the ethical principles outlined in the 1975 Declaration of Helsinki. All operations related to animal experiments were carried out in accordance with the National Institutes of Health (NIH) guidelines for the Care and Use of Laboratory Animals and were approved by the Ethics Committee of The First Affiliated Hospital of Zhengzhou University (Ethics Approval No. ZZU-LAC20210521[07]).

## Supplementary information

Supplementary Material

Full and uncropped western blots

## Data Availability

All data generated or analyzed during this study are included either in this article or in the Supplementary Material files. The transcriptome sequencing data presented in this study have been deposited in the GEO database, with the accession number GSE302256. The mass spectrometry proteomics data have been deposited to the ProteomeXchange Consortium via the PRIDE partner repository with the dataset identifier PXD066082.
