# Supplementary material for: USAG-1 aggravates renal ischemia‒reperfusion injury via promoting GPX4 degradation-induced ferroptosis
Source: Cell Death Dis. 2026 May 23;17(1):646. doi: 10.1038/s41419-026-08904-w (PMC13376362; doi:10.1038/s41419-026-08904-w)
Supplement: Supplementary file 1 — Supplementary Material [file 41419_2026_8904_MOESM1_ESM.docx]

**SUPPLEMENTARY MATERIAL**

**Supplemental Figure S1.**


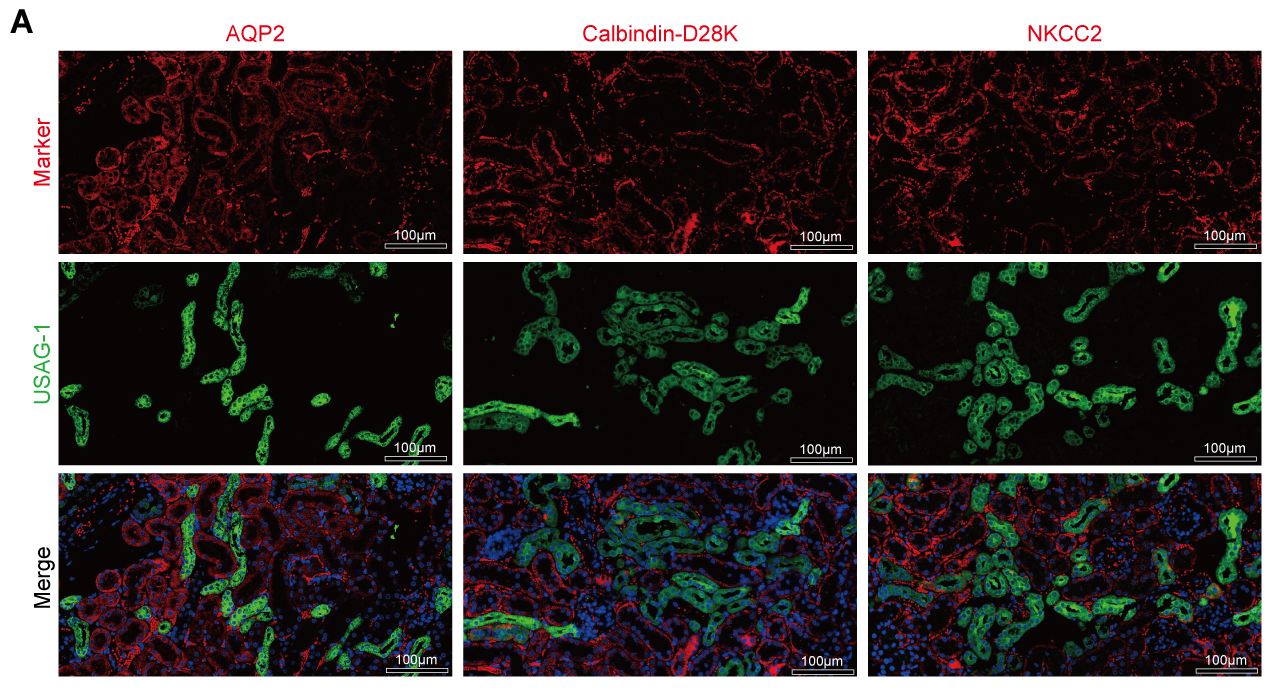


**Supplemental Figure S1. Additional co-immunostaining analyses of USAG-1 with markers of different nephron segments after 25 min of ischemia. A** Representative immunofluorescence images of USAG-1 co-staining with NKCC2, AQP2, and Calbindin-D28K in kidney sections after 25 min of ischemia (scale bar: 100 μm).

**Supplemental Figure S2.**


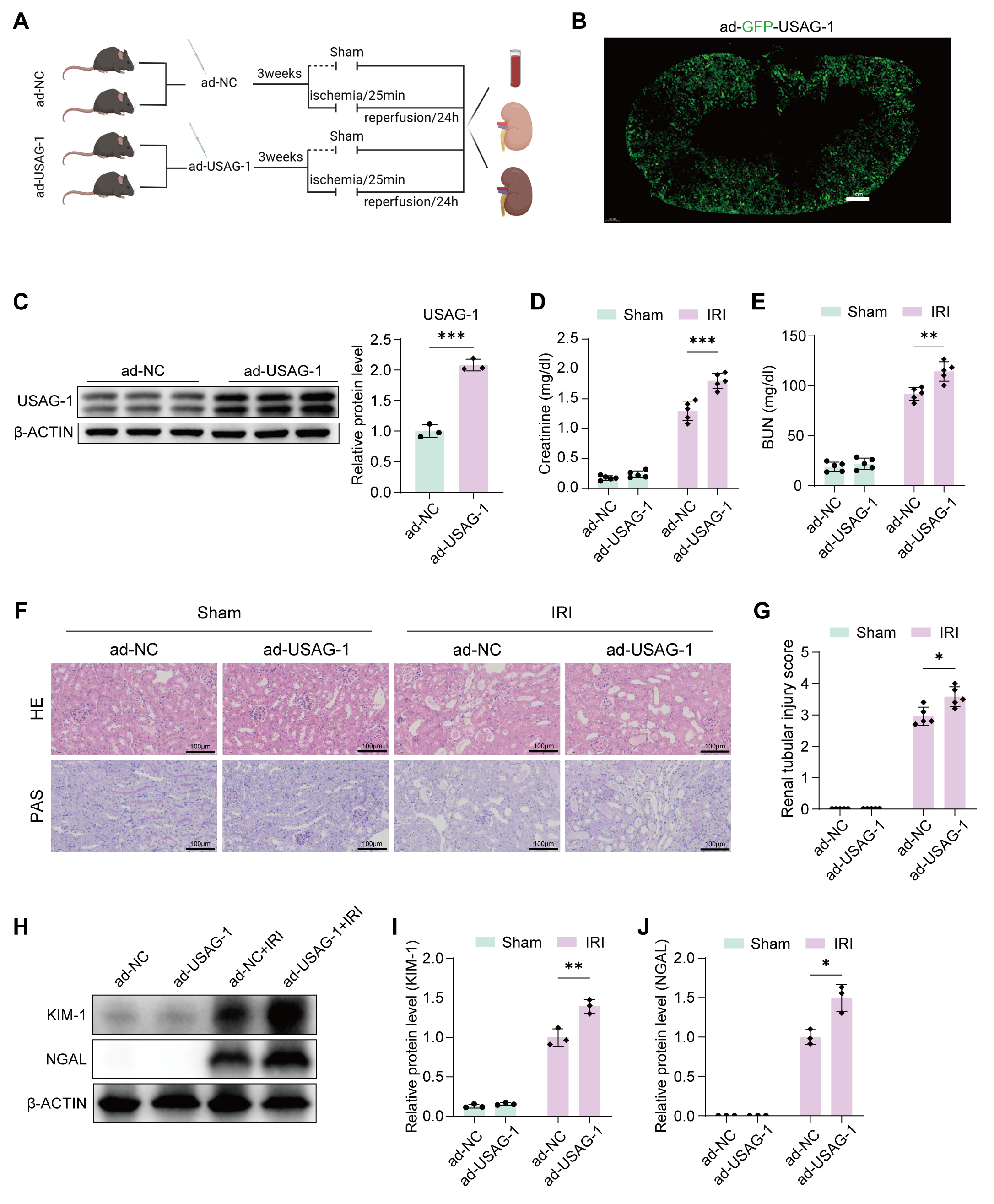


**Supplemental Figure S2. USAG-1 overexpression exacerbates renal injury following IRI. A** Schematic diagram of the experimental groups. **B** Representative GFP fluorescence images of kidney sections from mice with renal tubular epithelial cell-specific GFP-USAG-1 overexpression. **C** Western blot analysis and quantification of USAG-1 overexpression in kidneys from ad-NC and ad-USAG-1 mice under the indicated conditions. **D, E** Scr and BUN levels across in the indicated groups. **F, G** Representative H&E and PAS staining of kidney sections and tubular injury scores (scale bar: 50 μm; n = 5). **H–J** Western blot analysis and quantification of KIM-1 and NGAL expression in renal tissues (n = 3). ns : p > 0.05; * : p < 0.05; ** : p < 0.01; *** : p < 0.001.

**Supplemental Figure S3.**


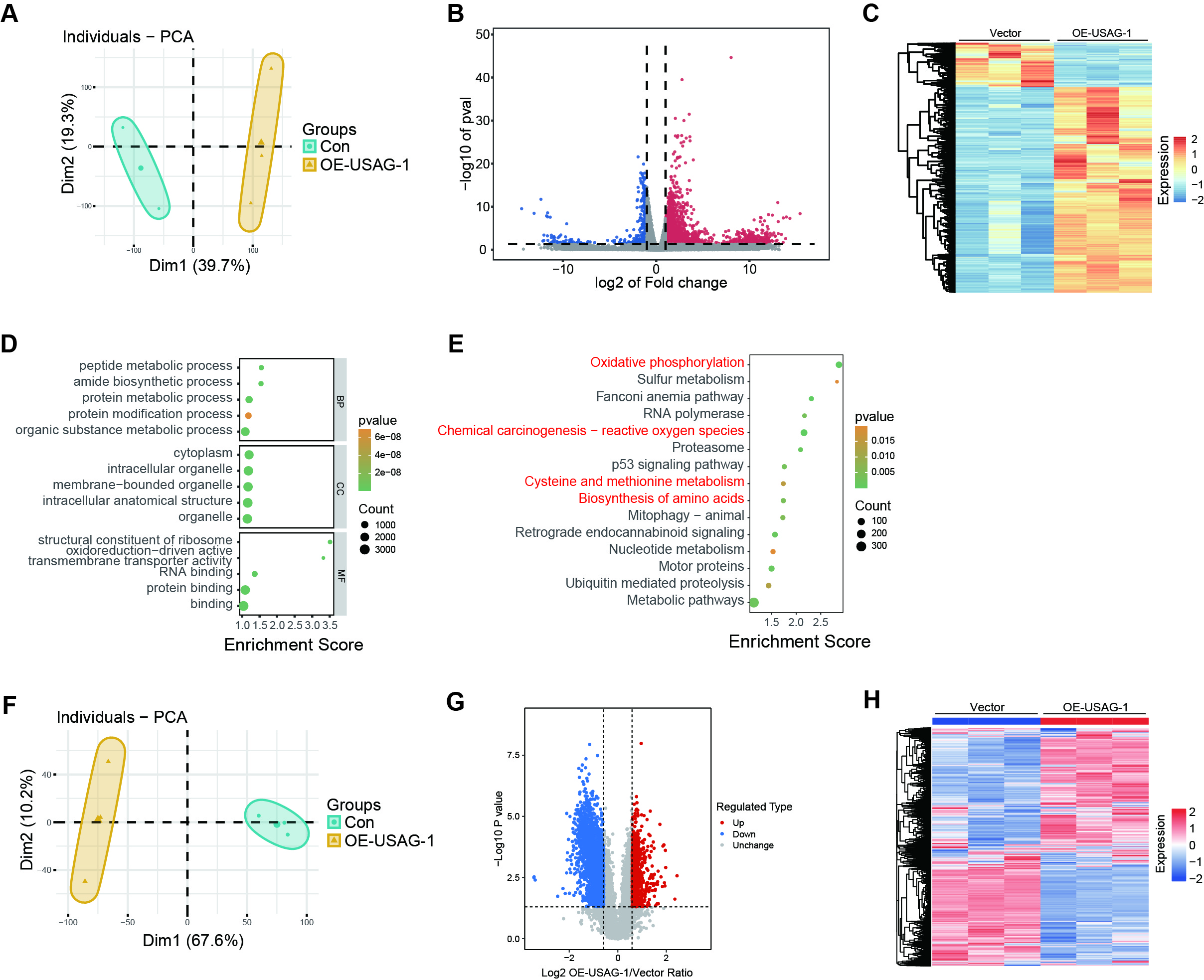


**Supplemental Figure S3. Transcriptomic and proteomic profiling following USAG-1 overexpression in HK-2 cells. A** Principal component analysis (PCA) of transcriptomic samples. **B, C** Volcano plot and heatmap showing differentially expressed genes after USAG-1 overexpression. **D, E** GO enrichment and KEGG pathway enrichment analyses of differentially expressed genes identified from the transcriptomic dataset. **F** Principal component analysis (PCA) of proteomic samples. **G, H** Volcano plot and heatmap showing differentially expressed proteins after USAG-1 overexpression.

**Supplemental Figure S4.**

**
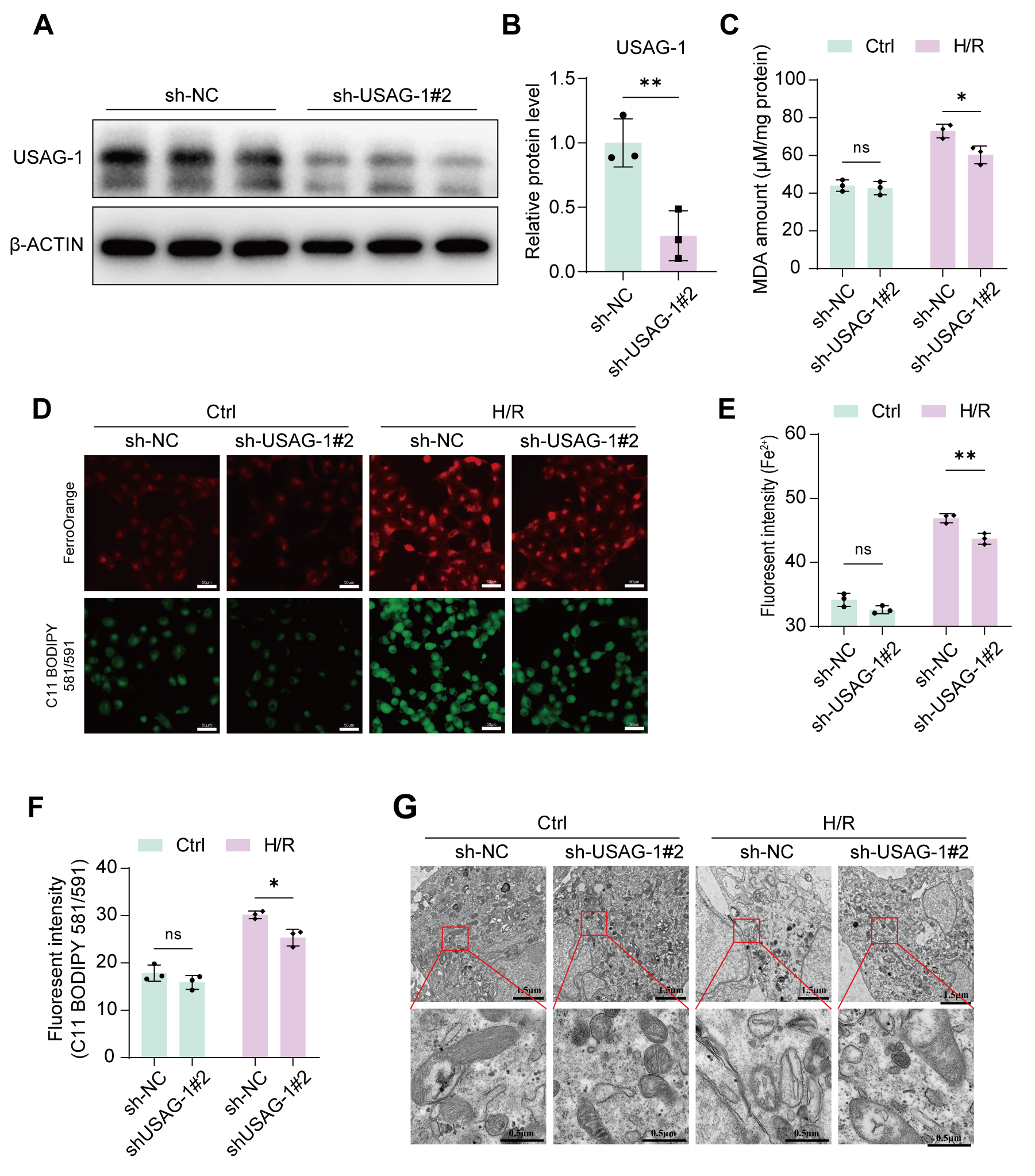
**

**Supplemental Fig. S4. Independent validation of USAG-1 knockdown-mediated suppression of ferroptosis in HK-2 cells using a second shRNA. A, B** Western blot analysis and quantification showing the knockdown efficiency of sh-USAG-1#2 in HK-2 cells. **C** Measurement of the levels of the lipid peroxidation product MDA in different groups after USAG-1 knockdown. **D–F** After USAG-1 knockdown with sh-USAG-1#2, intracellular Fe²⁺ and LPO levels were measured and quantified in the indicated groups. (scale bar: 50 μm; n = 3). **G** Representative TEM images showing mitochondrial ultrastructural changes in sh-NC and sh-USAG-1#2-transduced HK-2 cells under control or H/R conditions. ns : p > 0.05; * : p < 0.05; ** : p < 0.01; *** : p < 0.001.

**Supplemental Figure S5.**


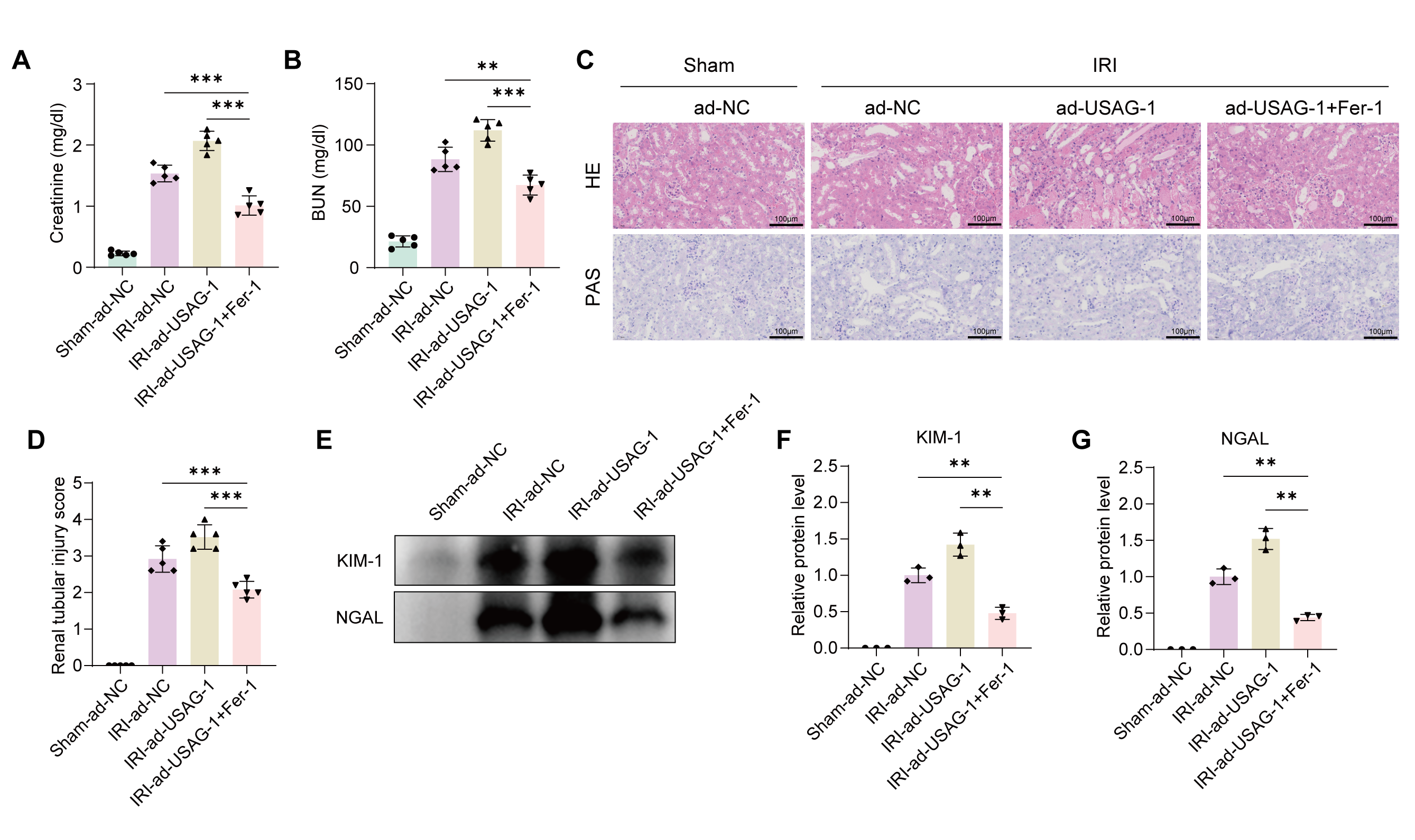


**Supplemental Figure S5. Fer-1 Reverses USAG-1**–**Induced Aggravation of IRI. A, B** Scr and BUN levels in the different groups. **C, D** Representative H&E and PAS staining of kidney sections and tubular injury scores (scale bar, 50 μm; n = 5). **E–G** Western blot analysis and quantification of KIM–1 and NGAL expression in renal tissues from different groups (n = 3). ns : p > 0.05; * : p < 0.05; ** : p < 0.01; *** : p < 0.001.

**Supplemental Figure S6.**

**Supplemental Figure S6. USAG-1 deficiency alleviates FA-induced AKI and ferroptosis. A** Schematic diagram of the experimental groups. **B, C** Scr and BUN levels in the indicated groups. **D, E** Representative HE, PAS, 4-HNE, and MDA immunohistochemical staining of kidney sections from the indicated groups and tubular injury scores (scale bar, 50 μm; n = 5). **F–H** Western blot analysis and quantification of KIM-1 and NGAL expression in whole-kidney lysates from the indicated groups (n = 3). **I** Measurement of LPO levels in renal tissues from the indicated groups (n = 5). **J** Measurement of total glutathione (GSH) levels in renal tissues from the indicated groups (n = 5). **K** Measurement of the GSH/GSSG ratio in renal tissues from the indicated groups (n = 5). **L** Representative TEM images showing mitochondrial morphology in proximal tubular cells from the indicated groups. ns : p > 0.05; * : p < 0.05; ** : p < 0.01; *** : p < 0.001.

**Supplemental Figure S7.**


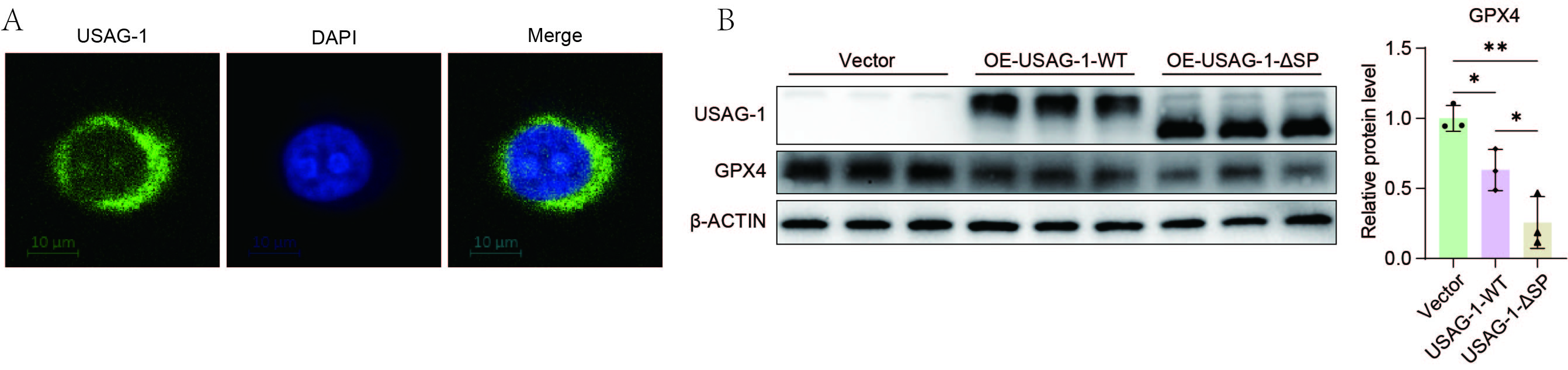


**Supplemental Figure S7. Cytoplasmic USAG-1 suppresses GPX4 protein expression. A** Immunofluorescence staining showing the subcellular localization of USAG-1 in HK-2 cells. **B** Western blot analysis and quantification of GPX4 protein expression in HK-2 cells transfected with USAG-1-WT or USAG-1-ΔSP. ns : p > 0.05; * : p < 0.05; ** : p < 0.01; *** : p < 0.001.

**Supplemental Figure S8.**


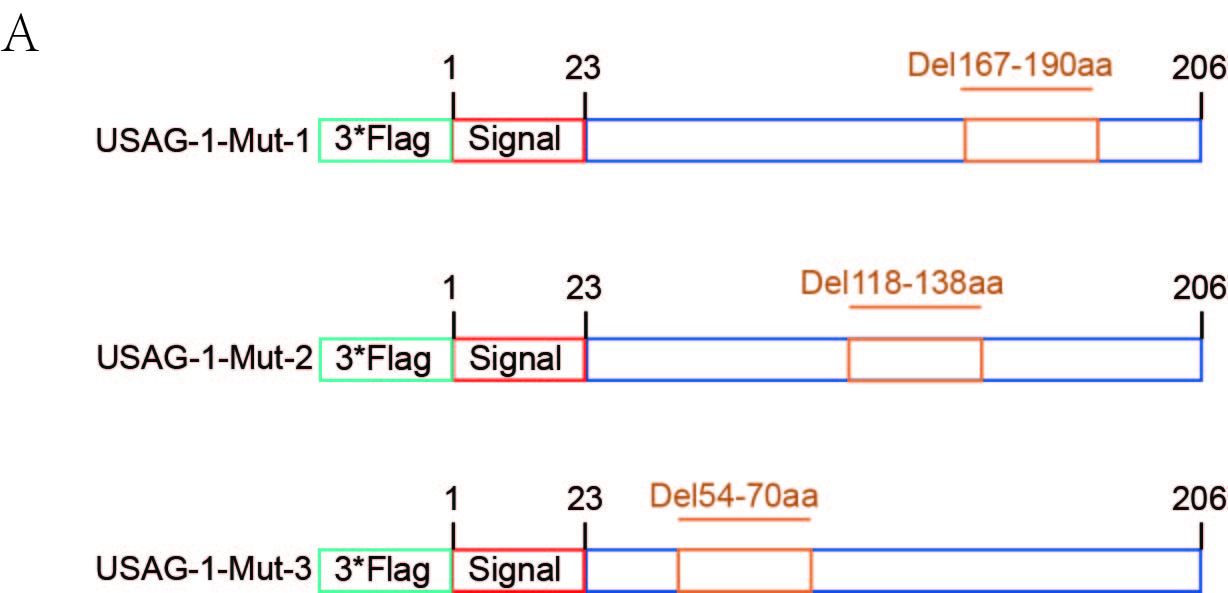


**Supplemental Figure S8. A** Mutation strategies for the construction of the USAG-1-Mut-1, USAG-1-Mut-2, and USAG-1-Mut-3 plasmids.

**Supplemental table 1**

| Data set | The union of these common elements |
| --- | --- |
| GSE43974  GSE186316  GSE30718 | *FOS* |
|  | *EGR1* |
|  | *ADAMTS1* |
|  | *CLDN8* |
|  | *TNFRSF12A* |
|  | *TMEM252* |
|  | *SOSTDC1* |

**Supplemental table 2** **Deceased-donor characteristics, stratified by recipients’ allograft function after transplantation**

| groups | ST (n = 34) | DGF (n = 36) | *P*-value |
| --- | --- | --- | --- |
| Age | 42.4 ± 12.6 | 45 ± 9 | 0.319 |
| Male/Female (n) | 27 / 7 | 33 / 3 | 0.262 |
| BMI(kg/m^2^) | 24.2 (21.7, 25.8) | 24.8 (23.1, 26.1) | 0.18 |
| Cause of death (n) | | | 0.088 |
| Brain trauma | 14 | 8 |  |
| Cerebrovascular disease | 20 | 28 |  |
| Terminal Scr (mg/dl) | 0.79 (0.58, 1.39) | 1.62 (0.75, 3.03) | 0.009 |

**Supplemental table 3** **Recipient characteristics, stratified by recipients’ allograft function**

| groups | ST (n = 52) | DGF (n = 52) | *P*-value |
| --- | --- | --- | --- |
| Age | 34.5 (31, 46) | 36 (31, 46.75) | 0.499 |
| Male/Female (n) | 24/28 | 33/19 | 0.076 |
| BMI (kg/m^2^) | 21.3 ± 2.8 | 21 ± 2.4 | 0.523 |
| Initial nephropathy (n) |  |  | 0.364 |
| Glomerulonephritis | 34 | 31 |  |
| Hypertensive nephropathy | 10 | 9 |  |
| IgA nephropathy | 5 | 10 |  |
| Diabetic Nephropathy | 0 | 1 |  |
| Other | 3 | 1 |  |
| Cold ischemia time (h) | 12.6 ± 2 | 14 ± 2.8 | 0.003 |
| Warm ischemia time (min) | 7 (5, 10) | 6 (5, 10) | 0.880 |
| HLA mismatch (n) | 4 (3, 4) | 4 (3, 5) | 0.132 |
| Remuzzi scores | 2 (2, 3) | 3 (2, 4) | 0.02 |
| Mode of dialysis |  |  | 0.669 |
| Hemodialysis/peritoneal dialysis | 44/8 | 46/6 |  |
| Dialysis time (m) | 17.5 (7.5, 32.25) | 20.5 (12, 39.75) | 0.194 |
| CNI |  |  | 0.352 |
| Tacrolimus/cyclosporine | 49/3 | 46/6 |  |

**Supplemental table 4** **Univariate and multivariate logistic regression analyses for the predictors of DGF**

|  | Univariate | | |  | Multivariate | | |
| --- | --- | --- | --- | --- | --- | --- | --- |
|  | *OR* | 95%CI | *P*-value |  | *OR* | 95%CI | *P*-value |
| Donor age | 1.035 | 0.998 - 1.073 | 0.065 |  |  |  |  |
| Donor sex | 0.355 | 0.115 - 1.093 | 0.071 |  |  |  |  |
| Donor BMI | 1.159 | 1.017 - 1.321 | 0.027 |  | 1.137 | 0.941 - 1.373 | 0.184 |
| Donor cause of death | 0.443 | 0.189 - 1.036 | 0.060 |  |  |  |  |
| Donor terminal SCr | 2.215 | 1.437 - 3.416 | 0.001 |  | 2.799 | 1.481 - 5.291 | 0.002 |
| Warm ischemia time | 0.982 | 0.833 - 1.159 | 0.982 |  |  |  |  |
| Cold ischemia time | 1.292 | 1.084 - 1.541 | 0.004 |  | 1.385 | 1.056 - 1.815 | 0.018 |
| Donor remuzzi scores | 1.606 | 1.052 - 2.453 | 0.028 |  | 1.679 | 0.856 - 3.292 | 0.132 |
| Recipient age | 1.015 | 0.976 - 1.057 | 0.456 |  |  |  |  |
| Recipient sex | 0.494 | 0.225 - 1.082 | 0.078 |  |  |  |  |
| Duration of dialysis before transplantation | 1.007 | 0.991 - 1.023 | 0.425 |  |  |  |  |
| HLA mismatch | 1.403 | 0.874 - 2.251 | 0.161 |  |  |  |  |
| Staining area of USAG-1 | 1.123 | 1.072-1.177 | 0.001 |  | 1.128 | 1.062-1.197 | 0.001^*^ |

**Supplemental table 5** **Primer sequences used for RT–qPCR in this study**

| Primer | Sequence |
| --- | --- |
| *USAG-1*-F (Human) | CAGCAACAGCACCCTGAATC |
| *USAG-1*-R (Human) | TCCTCCGATCCAGTTGGGAA |
| *USAG-1*-F (Mouse) | CCTGCCATTCATCTCTCTCTCA |
| *USAG-1*-R (Mouse) | CCGGGACAGGTTTAACCACA |
| *GPX4*-F (Mouse) | CGCCAAAGTCCTAGGAAACG |
| *GPX4*-R (Mouse) | AACTCGGAGCTGTTGCAGTA |
| *GAPDH*-F (Mouse) | TGGAAAGCTGTGGCGTGATG |
| *GAPDH*-R (Mouse) | TACTTGGCAGGTTTCTCCAGG |
| *GAPDH*-F (Human) | GGTGTGAACCATGAGAAGTATGA |
| *GAPDH*-R (Human) | GAGTCCTTCCACGATACCAAAG |
