# Supplementary material for: USAG-1 aggravates renal ischemia‒reperfusion injury via promoting GPX4 degradation-induced ferroptosis
Source: Cell Death Dis. 2026 May 23;17(1):646. doi: 10.1038/s41419-026-08904-w (PMC13376362; doi:10.1038/s41419-026-08904-w)

Full unedited gel for Figure1 H

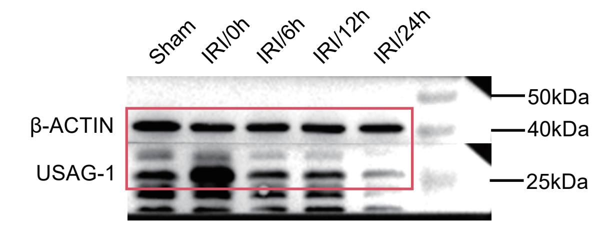

Full unedited gel for Figure1 J

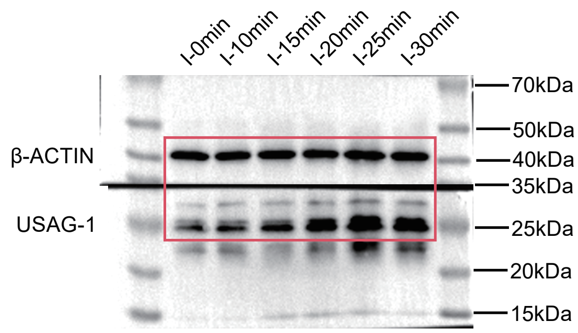

Full unedited gel for Figure2 D

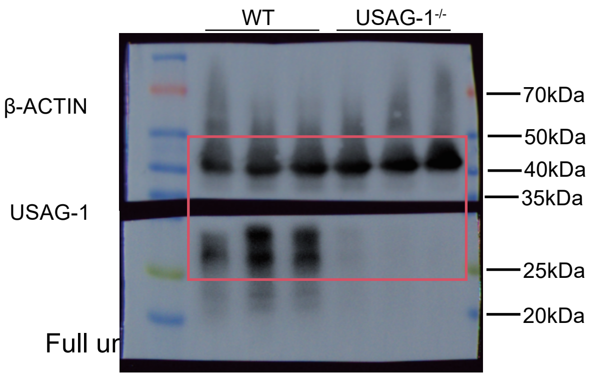

Full unedited gel for Figure2 I

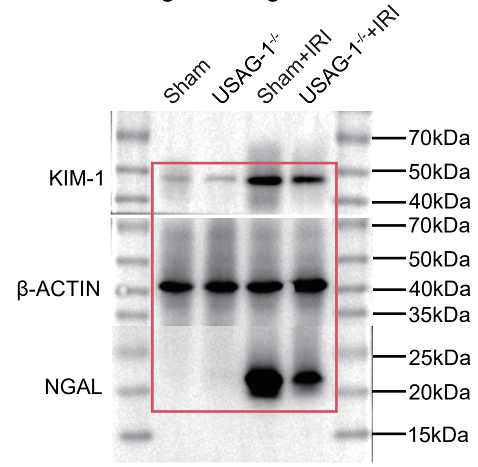

Full unedited gel for Figure3 D

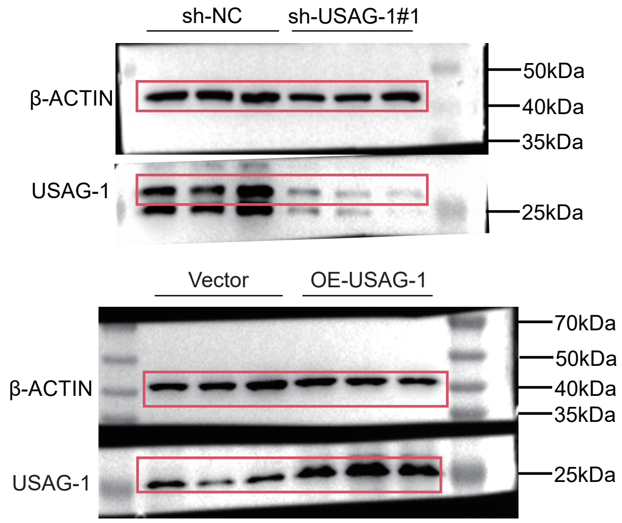

Full unedited gel for Supplemental Figure4 F

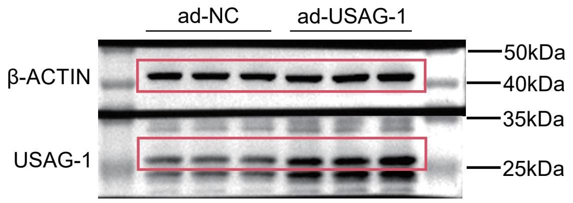

Full unedited gel for Figure5 C

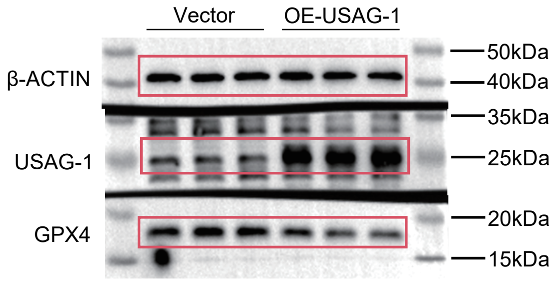

Full unedited gel for Figure5 F

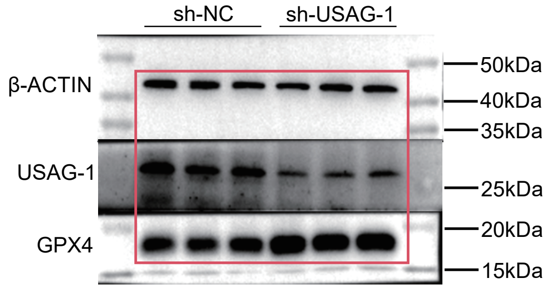

Full unedited gel for Figure5 I

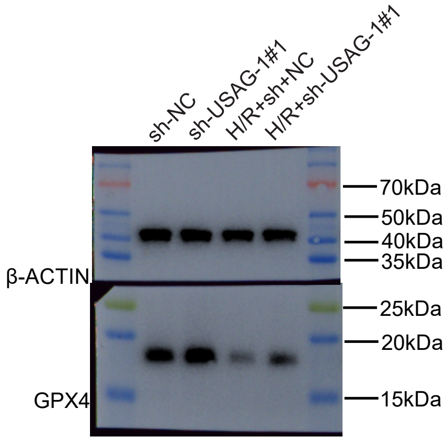

Full unedited gel for Figure5 K

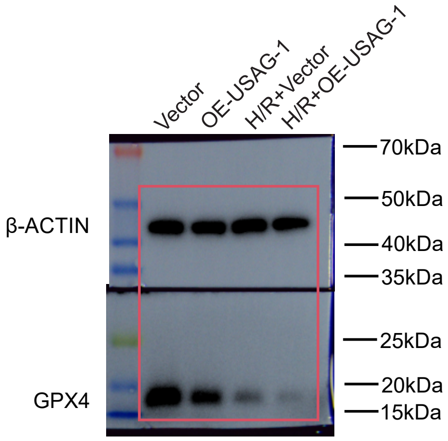

Full unedited gel for Figure6 A

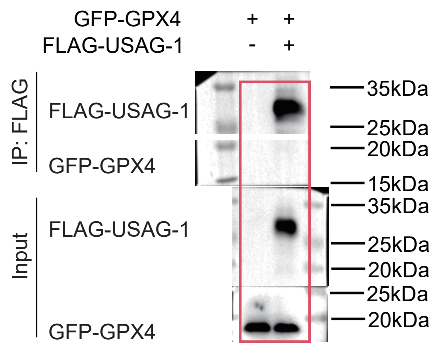

Full unedited gel for Figure6 D

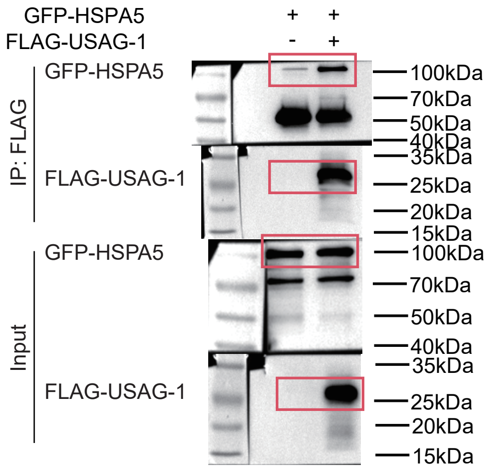

Full unedited gel for Figure6 F

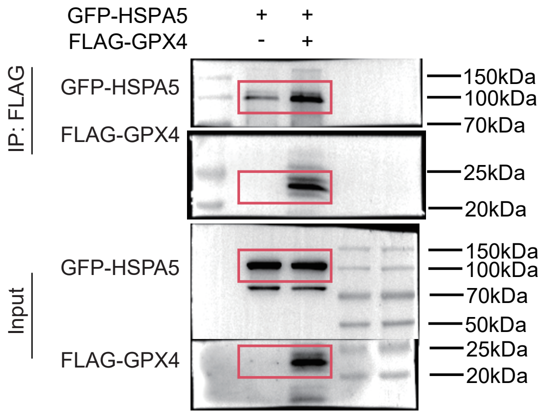

Full unedited gel for Figure6 G

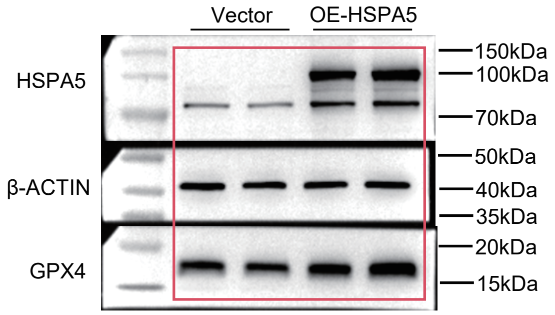

Full unedited gel for Figure6 H

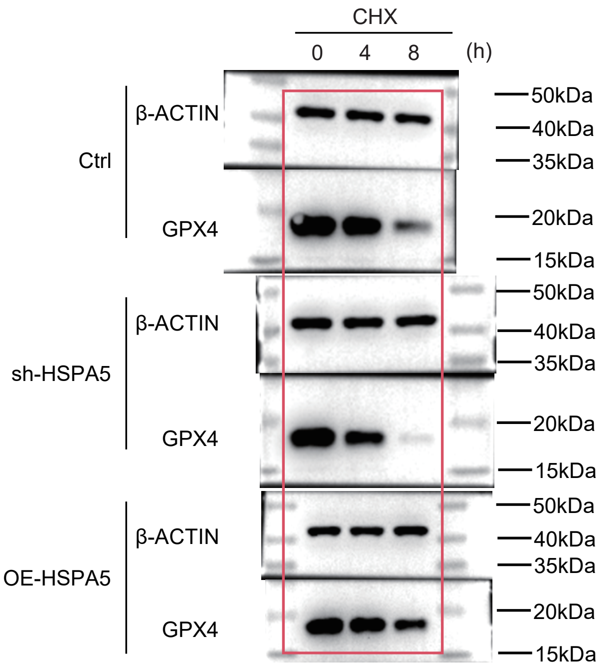

Full unedited gel for Figure6 I

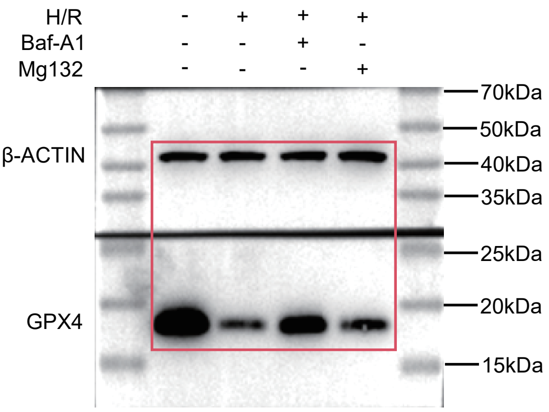

Full unedited gel for Figure6 J

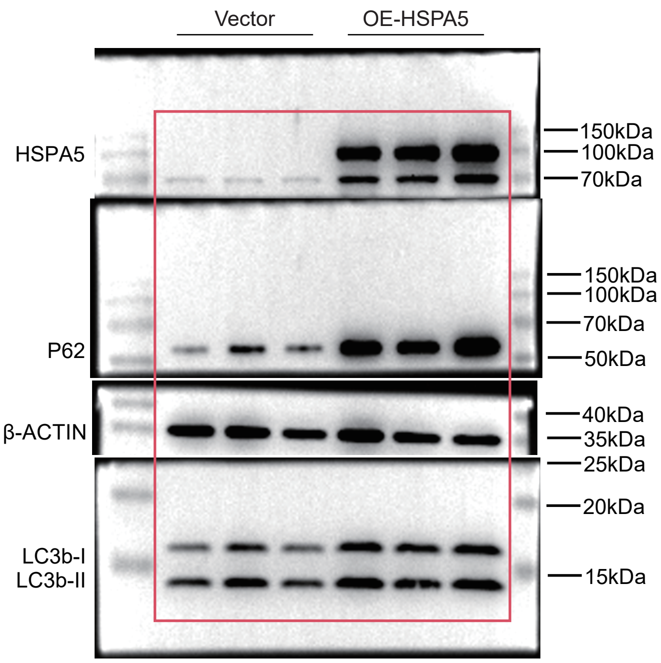

Full unedited gel for Figure6 K

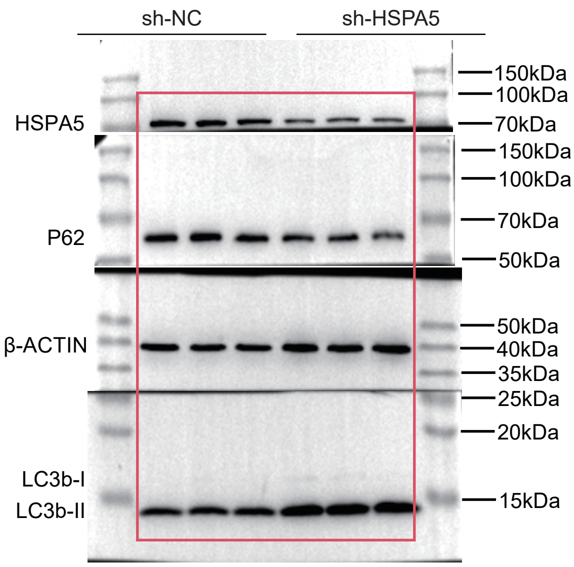

Full unedited gel for Figure6 L

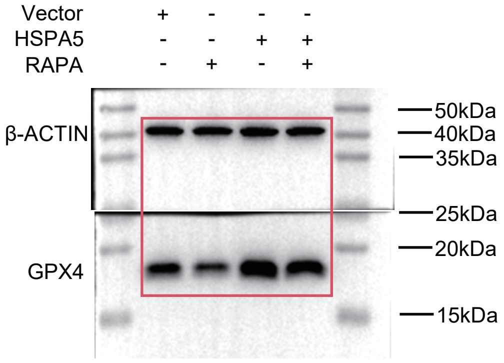

Full unedited gel for Figure6 M

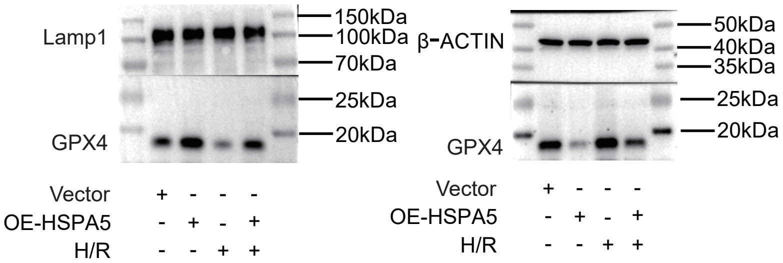

Full unedited gel for Figure7 A

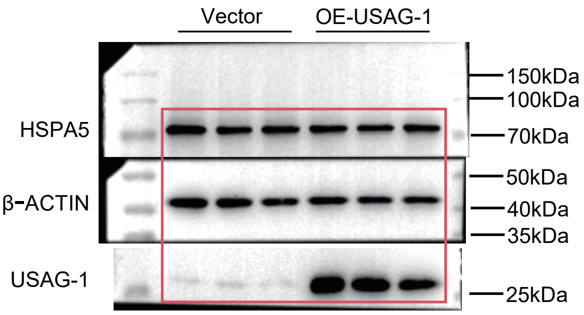

Full unedited gel for Figure7 C

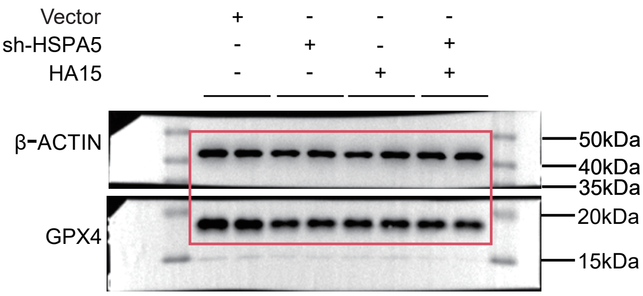

Full unedited gel for Figure7 B

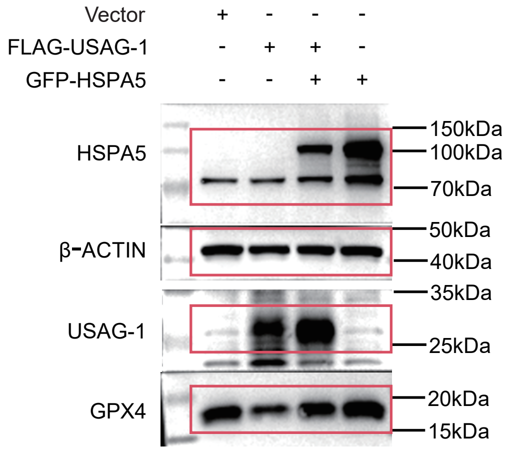

Full unedited gel for Figure7 D

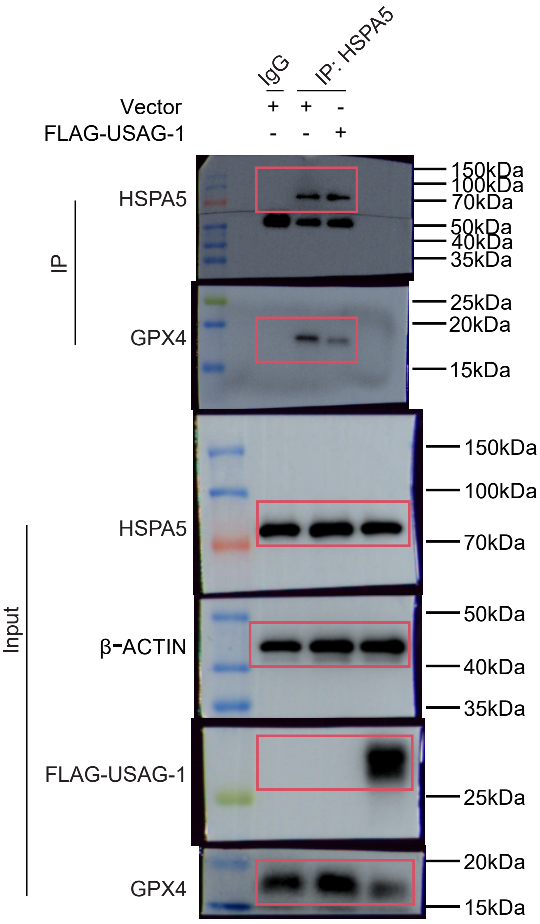

Full unedited gel for Figure7 F

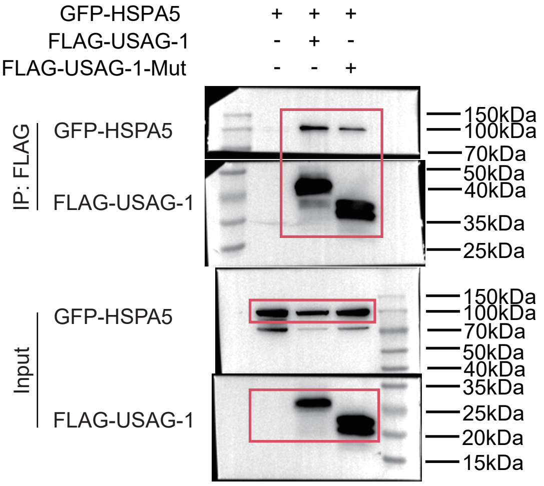

Full unedited gel for Figure7 G

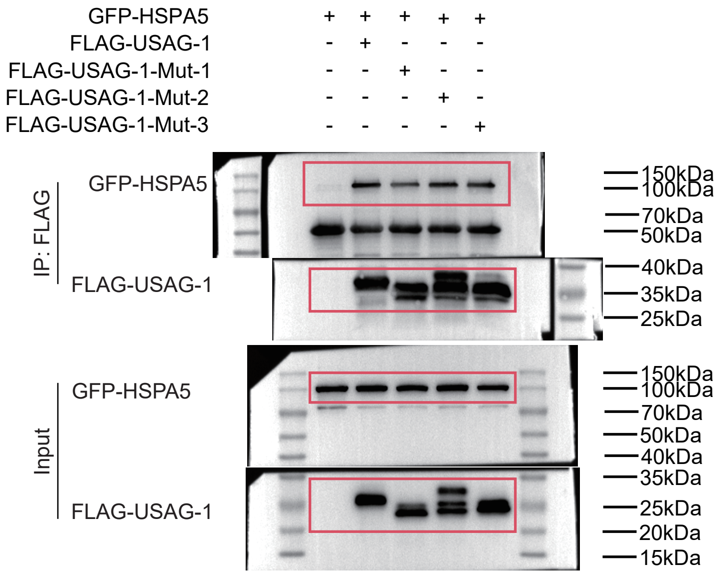

Full unedited gel for Figure7 H

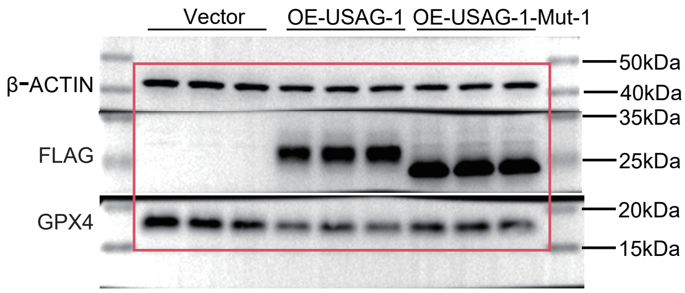

Full unedited gel for Figure7 I

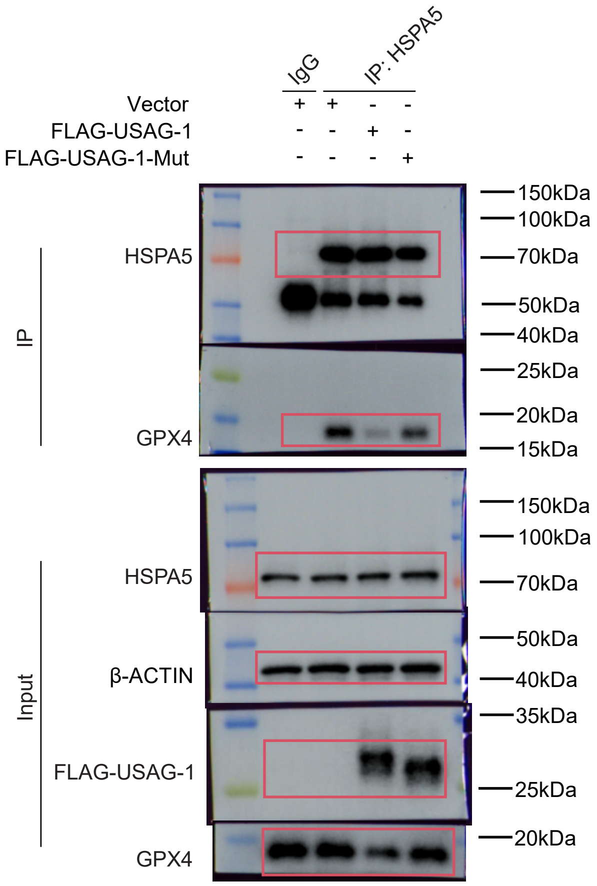

Full unedited gel for Figure7 J

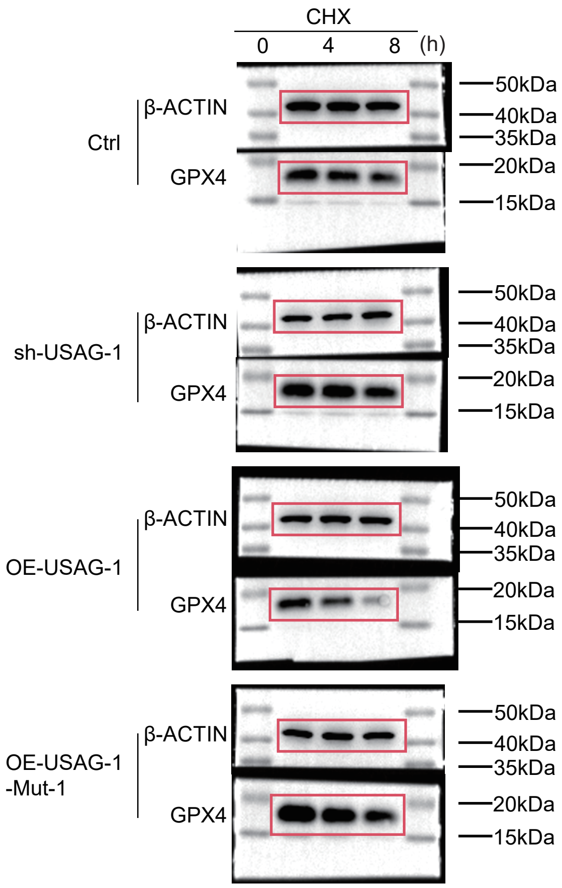

Full unedited gel for Figure8 B

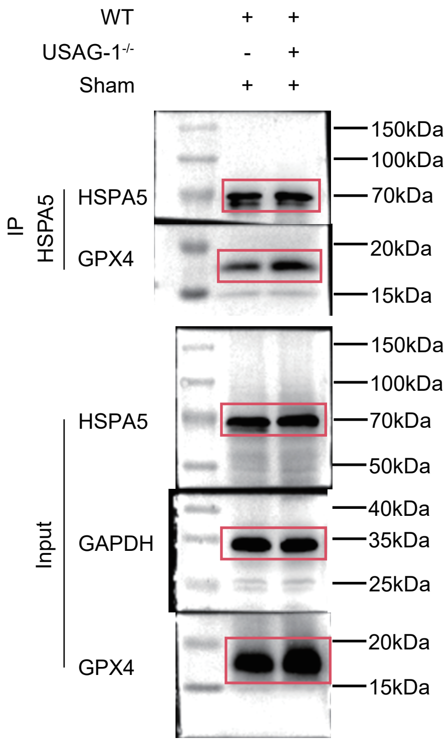

Full unedited gel for Figure8 C

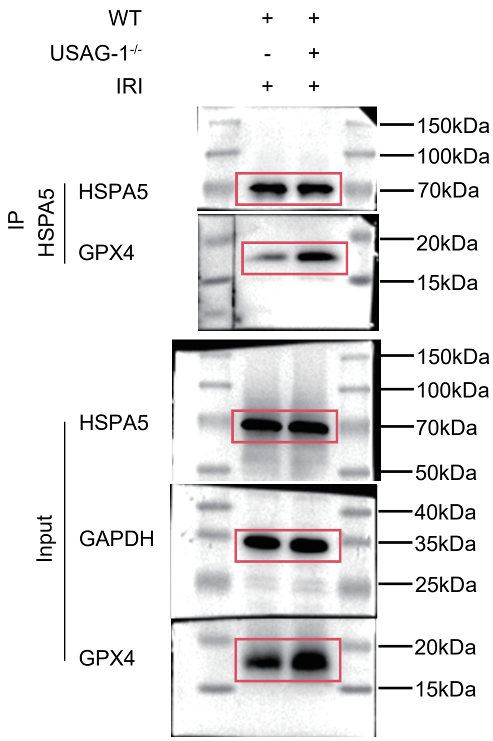

Full unedited gel for Supplemental Figure2 J

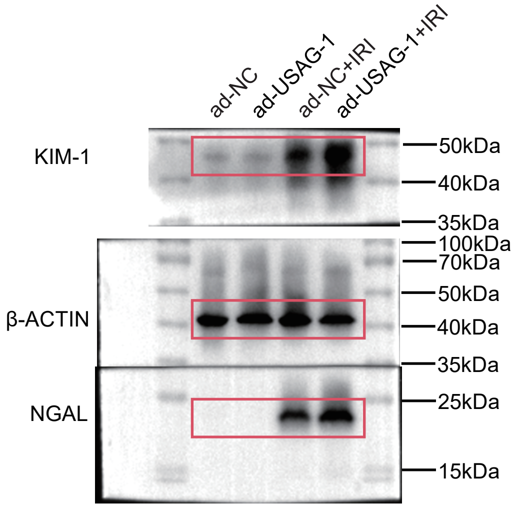

Full unedited gel for Supplemental Figure4 A

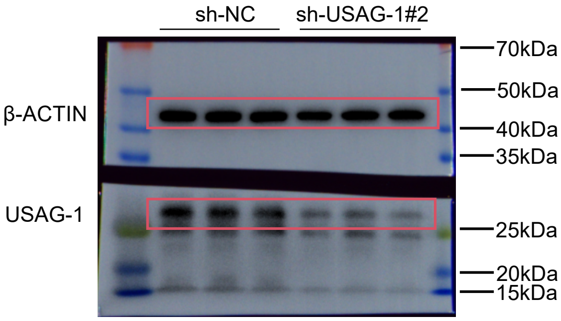

Full unedited gel for Supplemental Figure5 C

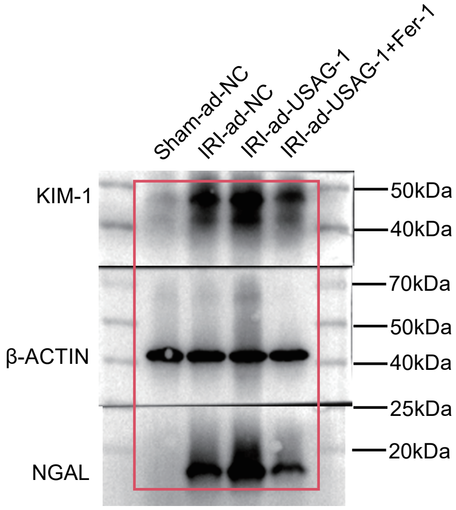

Full unedited gel for Supplemental Figure6 F

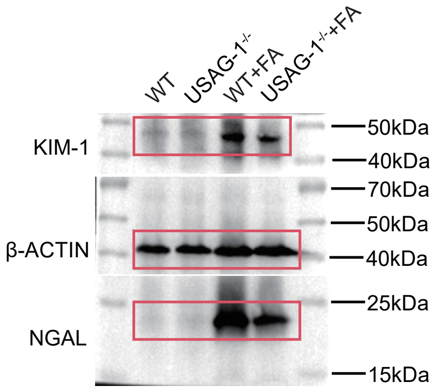

Full unedited gel for Supplemental Figure7 B

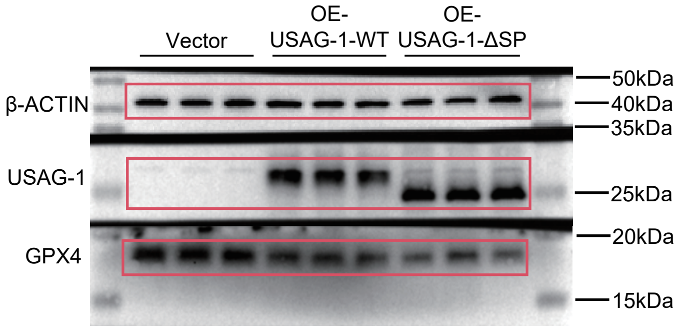

Supplement: Supplementary file 2 — Full and uncropped western blots [file 41419_2026_8904_MOESM2_ESM.pdf]
